# Supplementary material for: Helical reconstruction of VP39 reveals principles for baculovirus nucleocapsid assembly
Source: Nat Commun. 2024 Jan 4;15:250. doi: 10.1038/s41467-023-44596-y (PMC10767040; doi:10.1038/s41467-023-44596-y)
Supplement: Supplementary file 1 — Supplementary Information [file 41467_2023_44596_MOESM1_ESM.pdf]

SUPPLEMENTARY INFORMATION

Helical reconstruction of VP39 reveals principles for baculovirus nucleocapsid assembly

Friederike M. C. Benning[1,2], Simon Jenni[3], Coby Y. Garcia[1,4], Tran H. Nguyen[1], Xuewu Zhang[5,6], Luke H. Chao[1,2,*]

[1]Department of Molecular Biology, Massachusetts General Hospital, Boston, MA 02114, USA

[2]Department of Genetics, Harvard Medical School, Boston, MA 02115, USA

[3]Department of Biological Chemistry and Molecular Pharmacology, Harvard Medical School, Boston, MA 02115, USA

[4]Harvard College, Cambridge, MA 02138, USA

[5]Department of Biophysics, University of Texas Southwestern Medical Center, Dallas, TX 75390, USA

[6]Department of Pharmacology, University of Texas Southwestern Medical Center, Dallas, TX 75390, USA

*Correspondence: chao@molbio.mgh.harvard.edu

Supplementary Fig. 1: Data processing scheme.

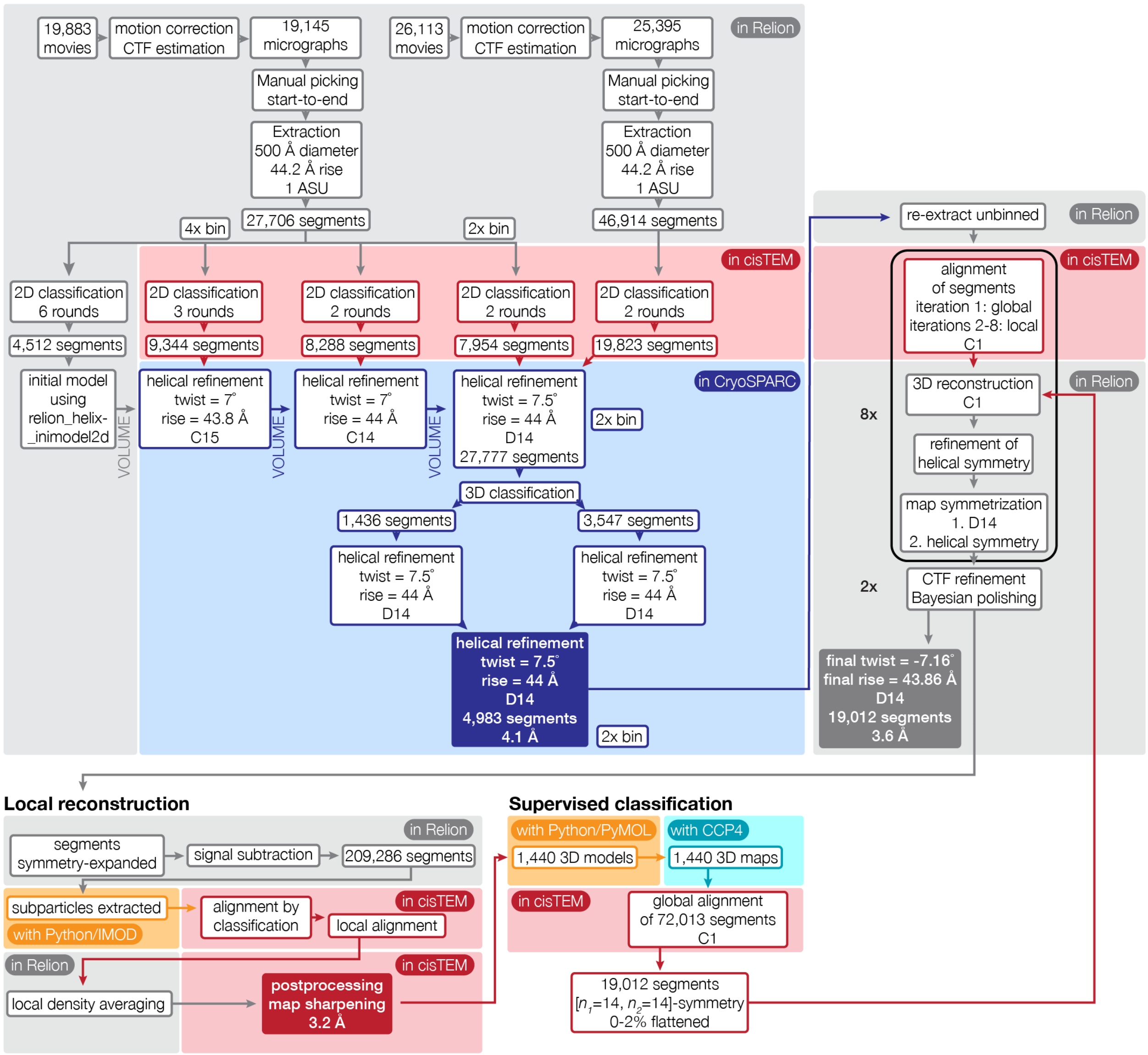

Depiction of the data processing steps used for generating the AcMNPV VP39 reconstruction and segment analysis by supervised classification. Colored boxes indicate the different programs used: Relion (gray), cisTEM (red), CryoSPARC (blue), Python/IMOD and Python/PyMOL (yellow) and CCP4 (cyan).

Supplementary Fig. 2: Variation in tube diameter.

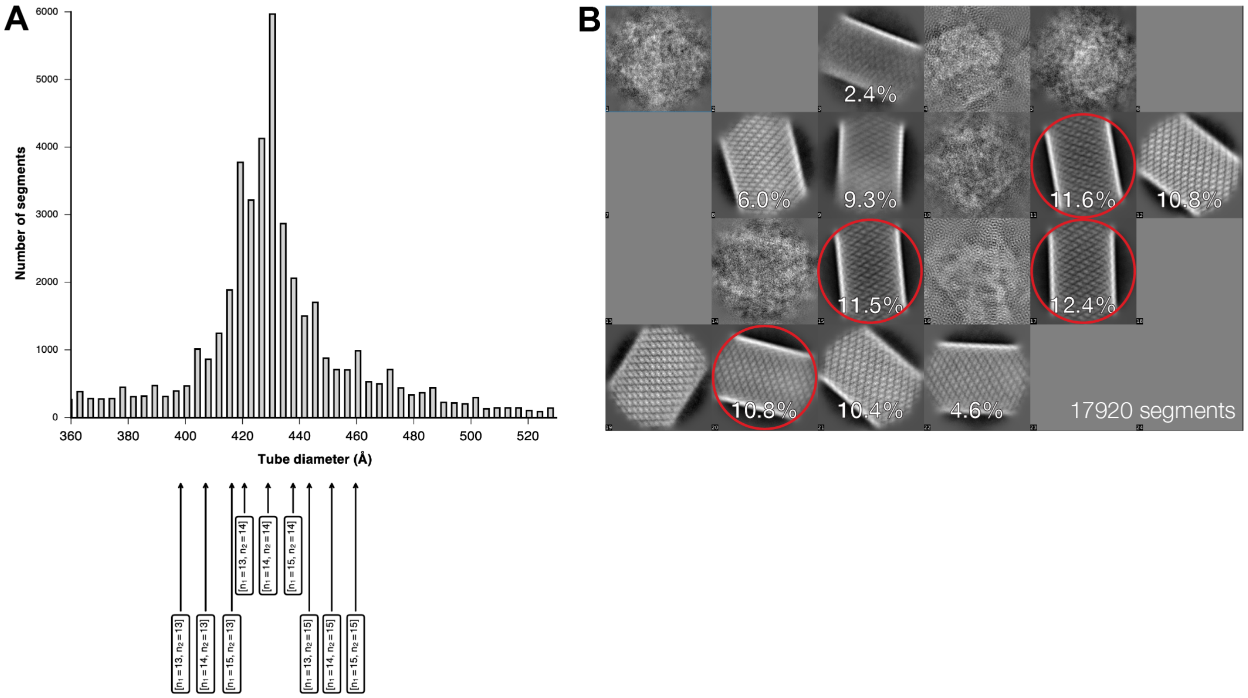

(A) Distribution of segment widths of 72,013 extracted segments. Segment widths were measured by aligning the helical axis based on the zero layer line signal of the power spectrum, projecting the density on a line and then measuring the distance between the two top peaks. Histogram bins with tube diameters <360 Å or >530 Å are not shown, as these measured widths were likely wrong, because corresponding segments were extracted close to the tube ends or affected by contamination in the micrographs. The arrows at the bottom of the histogram indicate the diameters of helixes with different symmetries (see Fig. 5). Source data are provided as a Source Data file.

(B) 2D classification of 17,920 segments (2x binned, 1.65 Å/px) in cisTEM[1] detects tube diameter variation to a certain extent. Selected class averages for further processing are circled in red.

Supplementary Fig. 3: Determination of helical symmetry parameters by Fourier-Bessel indexing.

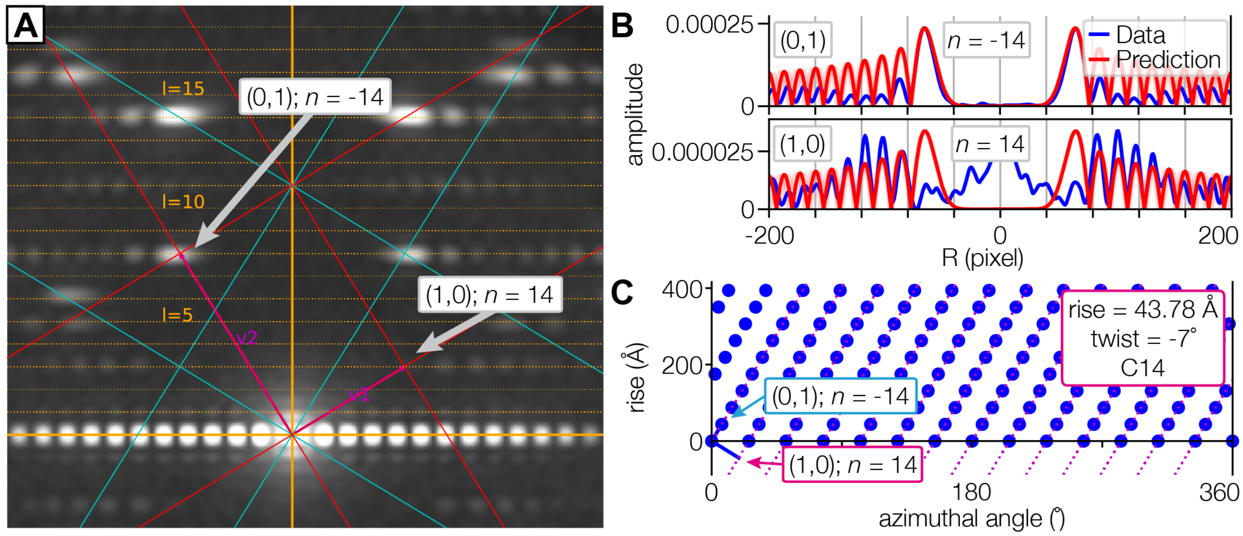

(A) Average power spectrum of all segments of the final [n_1=14, n_2=14]-reconstruction. Layer lines are shown as dashed lines in orange. Unit vectors $\mathbf{v_1}$ and $\mathbf{v_2}$ (pink) were used to generate a 2D lattice (red) consistent with peaks in the power spectrum. The mirror lattice is depicted in cyan.

(B) Amplitude diagrams for measured (blue) and predicted (red) data on layer line *l*=8 (for vector (0,1)) and on layer line *l*=3 (for vector (1,0)) of the power spectrum in (A) for Bessel order *n*=14.

(C) Real-space 2D lattice calculated from the Fourier-space lattice in (A) with the corresponding helical symmetry parameters.

Supplementary Fig. 4: Fourier shell correlations (FSCs) for the helical and local reconstructions, respectively, at different stages of cryo-EM data processing.

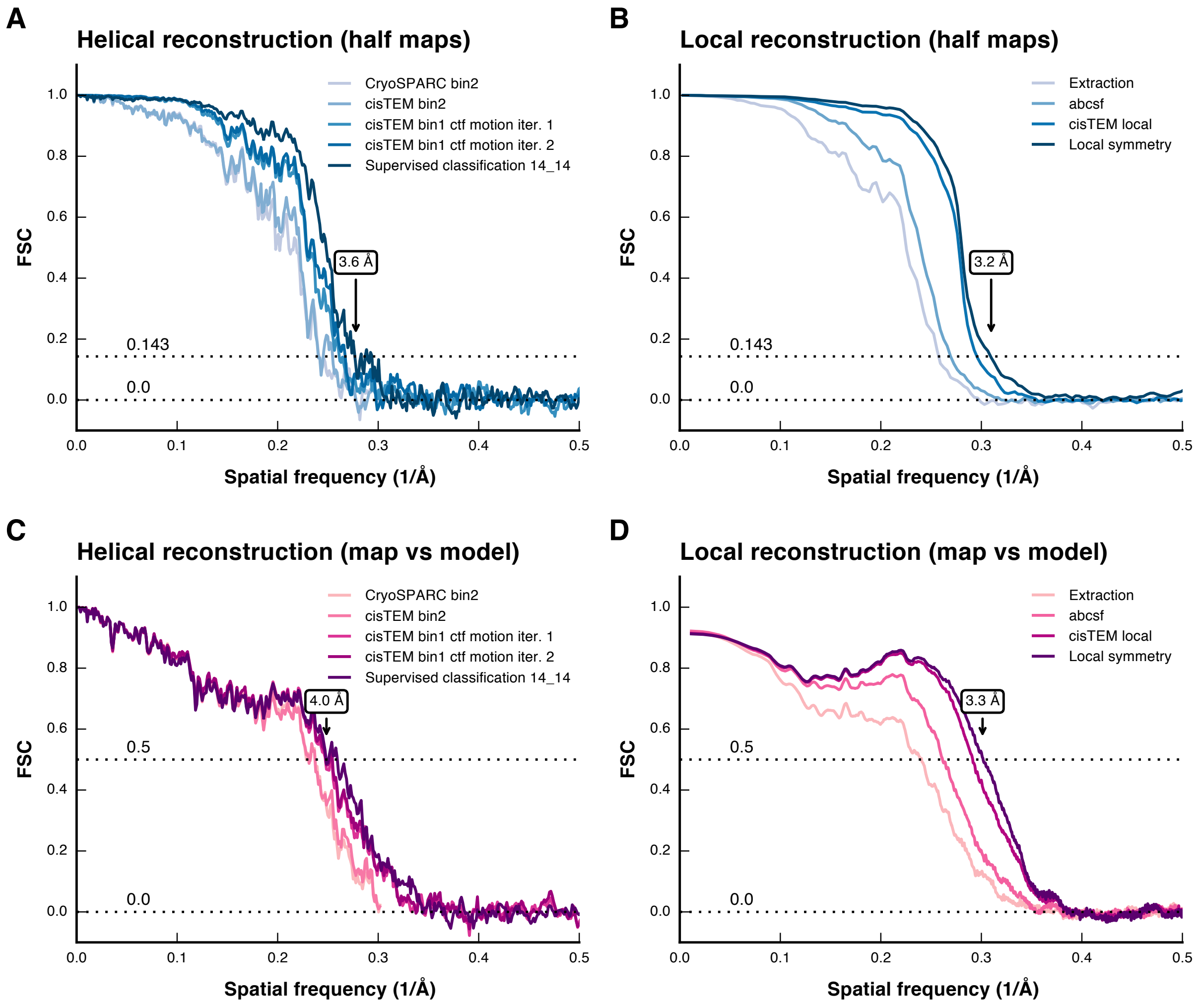

(A) FSC curves between half maps of the helical reconstruction. Correlation was calculated with e2proc3d.py from EMAN2[2] after masking the symmetrized half maps of the helical reconstructions. The final nominal resolution of 3.6 Å is indicated.

(B) FSC curves between half maps of the local reconstruction. Correlation was calculated with e2proc3d.py from EMAN2[2] after masking the half maps of the local reconstructions. The final nominal resolution of 3.2 Å is indicated.

(C) FSC curves between full maps and the final refined model of the helical reconstruction. The final refined model of a VP39 dimer was placed into the full map, rigid-body refined, and symmetry expanded to generate the full helix. We used the programs sfall and fftbig from CCP4[3] to calculate model structure factors and a model map. Correlation was then calculated with e2proc3d.py from EMAN2[2] after masking the symmetrized full map of the helical reconstructions and the model map, respectively. The final nominal resolution of 4.0 Å is indicated.
(D) FSC curves between full maps and the final refined model of the local reconstruction. Correlation was calculated with phenix.mtriage[4]. The final nominal resolution of 3.3 Å is indicated.
Source data for all FSC curves are provided as a Source Data file.

Supplementary Fig. 5: Depiction of local resolution of the cryo-EM map and B factors of the model.

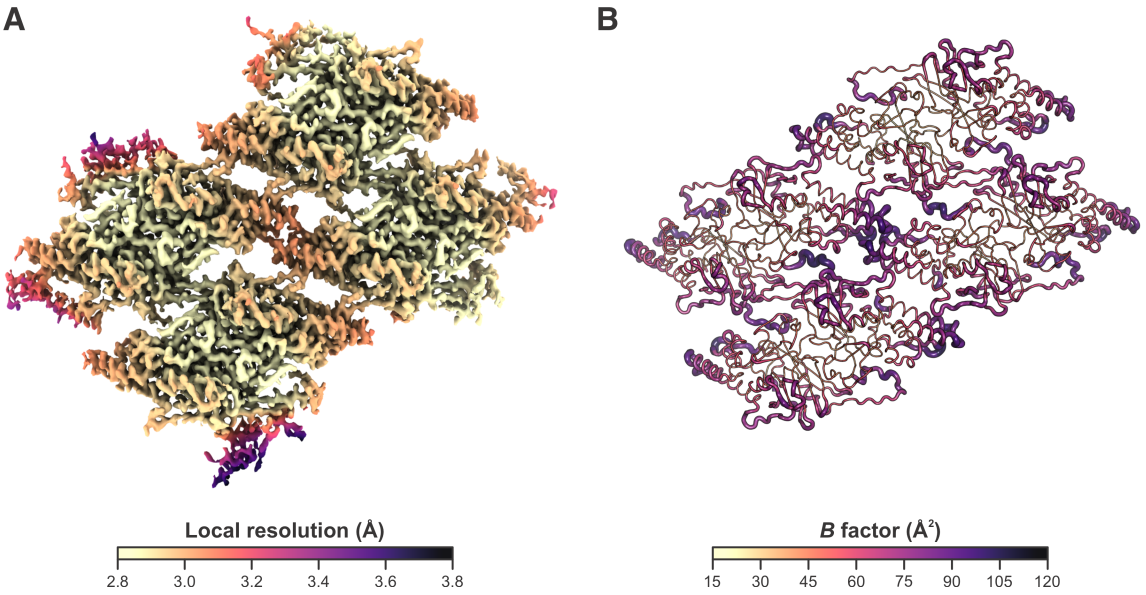

(A) Estimated local resolution mapped onto the local reconstruction of four adjacent dimers. Resolution estimates are color-coded from light (high resolution) to dark (low resolution).

(B) Model of four adjacent dimers color coded by its refined B factors from light (low) to dark (high).

Supplementary Fig. 6: Close-up view of the cryo-EM density map after local reconstruction with a nominal resolution of 3.2 Å.

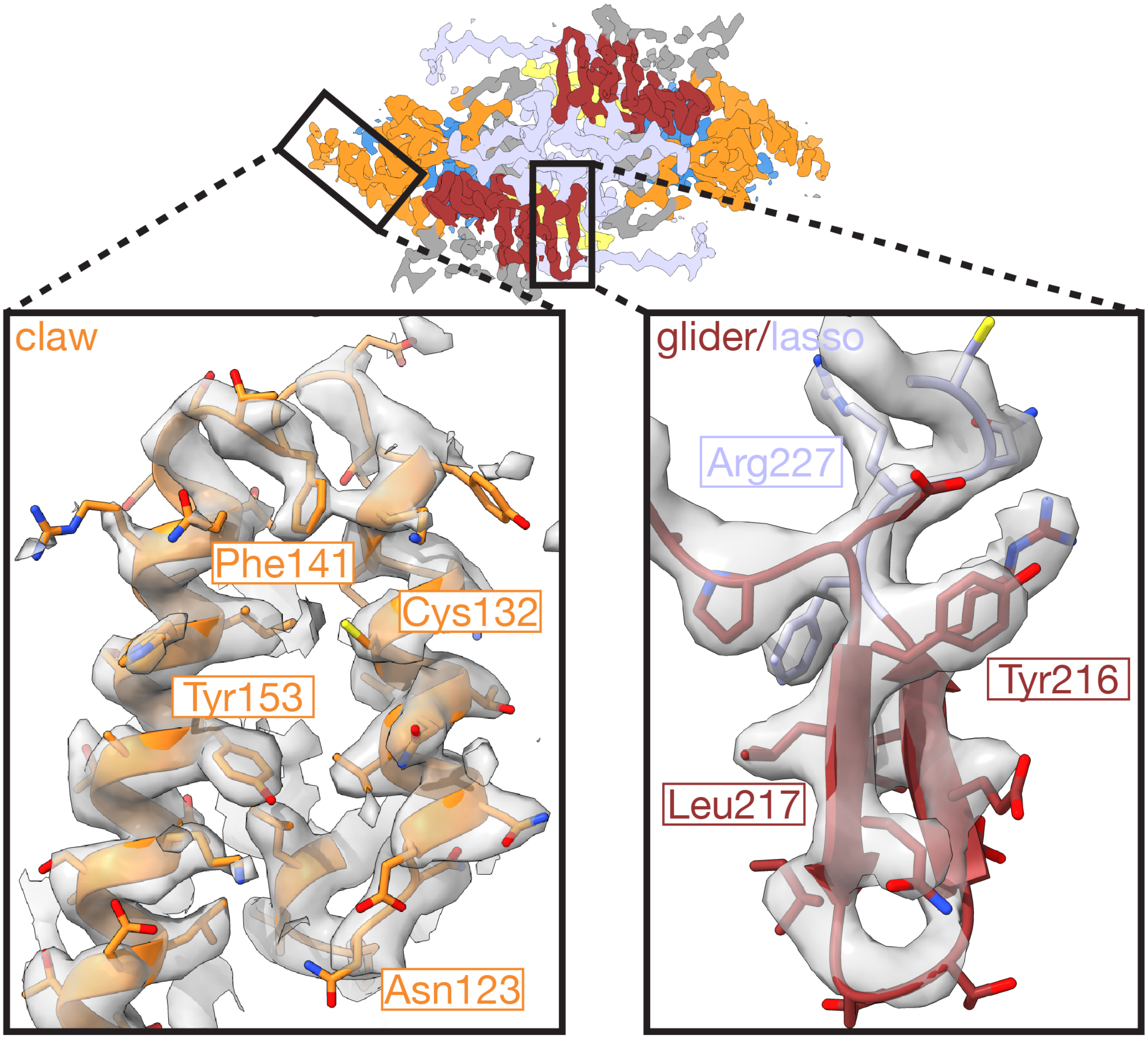

Residues 120-163 of the claw region (panel left; orange) and residues 213-229 of the glider and lasso region (panel right; red for glider and violet for lasso) are depicted.

Supplementary Fig. 7: Zn-coordination site in the AcMNPV VP39 ZF region.

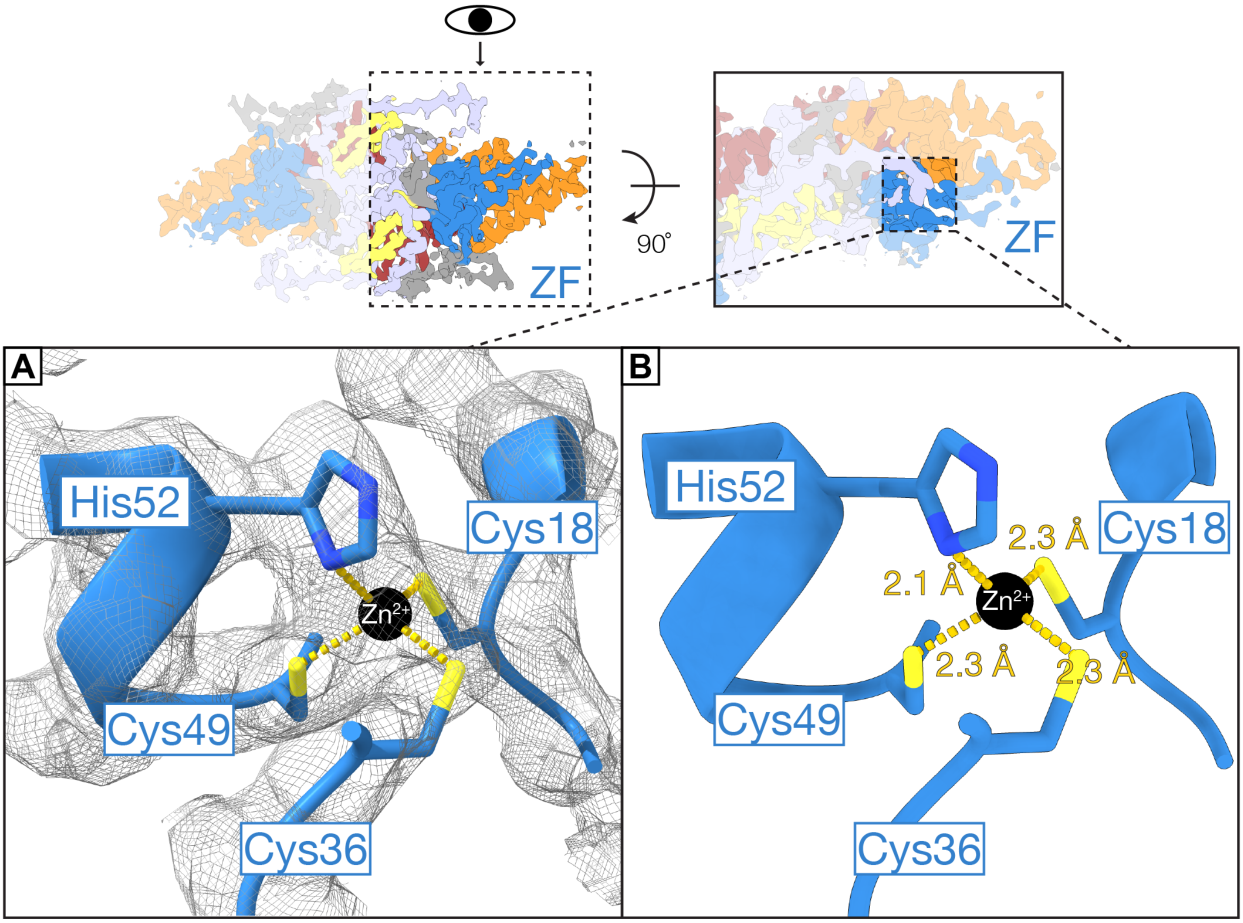

(A) Close-up view of the cryo-EM density at the Zn-coordination site. Cysteine 18, cysteine 36, cysteine 49 and histidine 52 coordinate a Zn^{2+} ion (black sphere).

(B) Close-up view of the Zn-coordination site with nitrogen/sulphur atom-Zn^{2+} distances shown. Distances were measured in ChimeraX[5].

Supplementary Fig. 8: Electrostatic surface potential of the AcMNPV VP39 capsid.

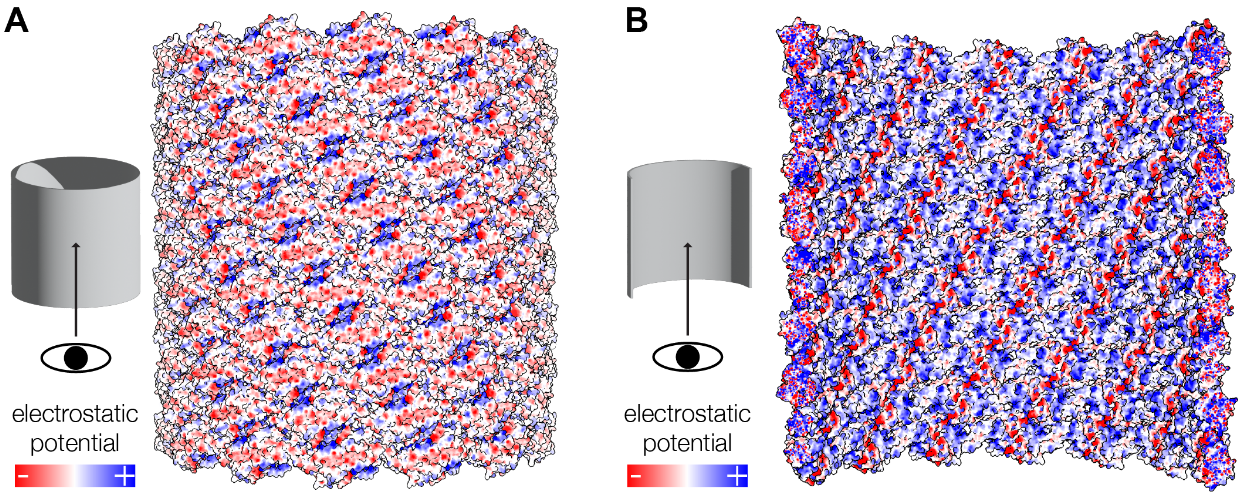

(A) Exterior view of the VP39 capsid. Surface representation of the VP39 model, which was fitted in the 3.6-Å cryo-EM capsid reconstruction. Surface is colored according to electrostatic potential from negative (red) to positive (blue).

(B) Luminal view of the VP39 capsid. Model fitting and electrostatic potential coloring were performed using ChimeraX[5].

Supplementary Fig. 9: Two reduced cysteine pairs at the lateral inter-subunit interface.

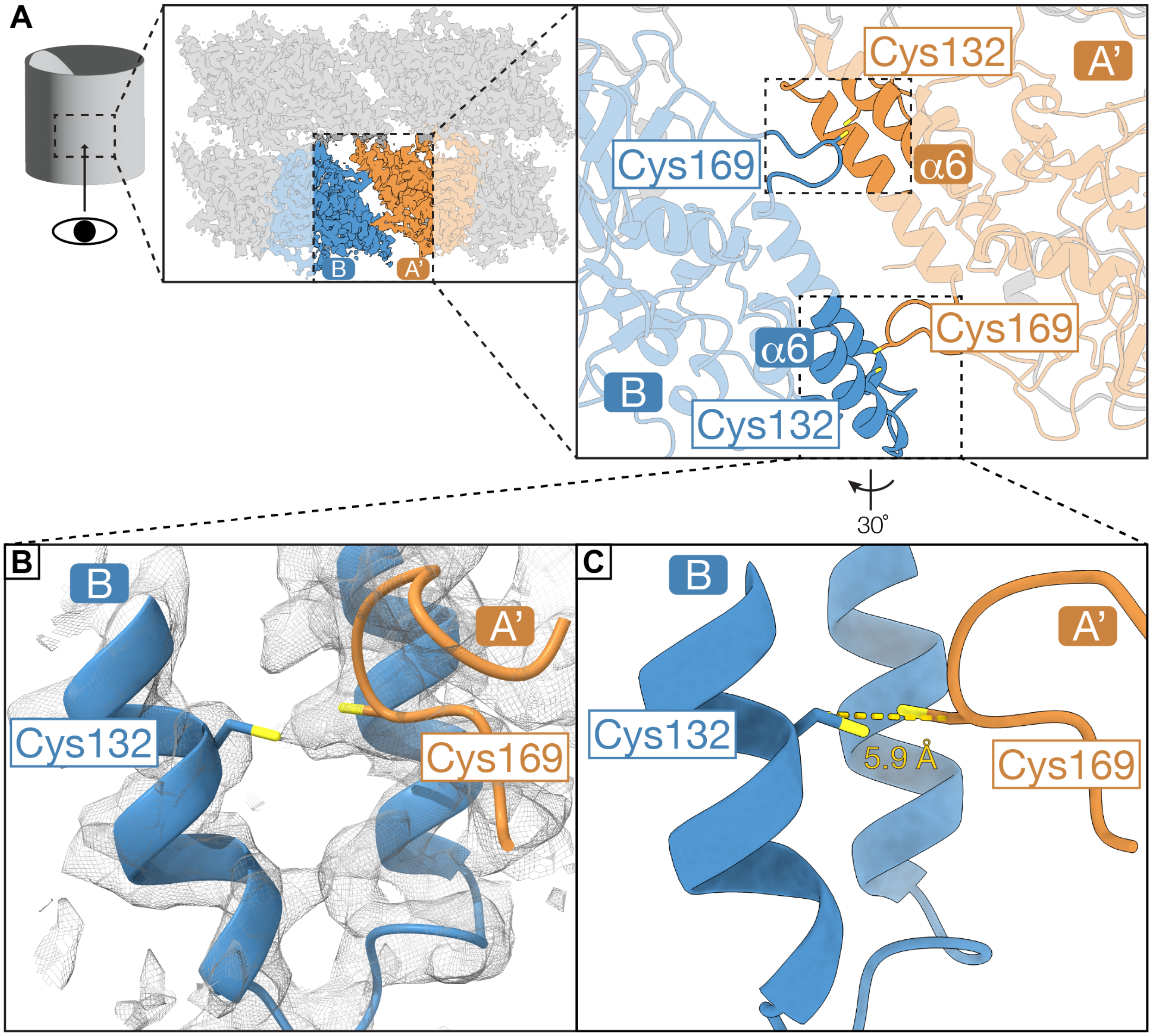

(A) Schematic overview of the location of the two potential disulfide pairs, viewed from the exterior side of the capsid. The four cysteines are located on the claw regions of two laterally adjacent subunits B (blue) and A' (ocher).

(B) Close-up view of the cryo-EM density around the two reduced cysteine residues cysteine 132 in monomer B and cysteine 169 in monomer A'.

(C) Close-up view of one of the two candidate disulfide pairs with Cα-Cα distance shown. Distance was measured in ChimeraX[5].

Supplementary Fig. 10: Alignment of baculoviral VP39 sequences.

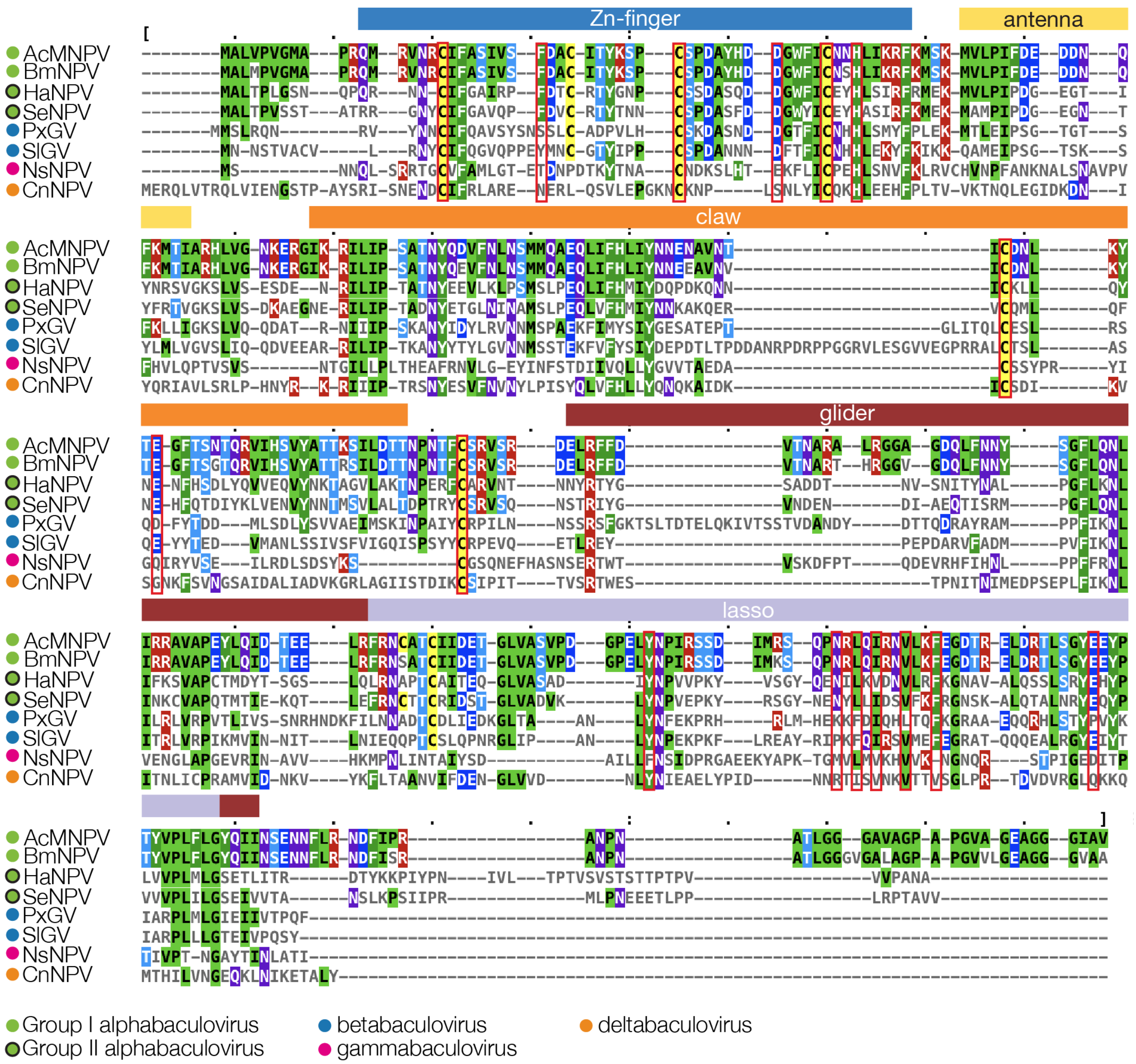

8 selected VP39 sequences are shown from an alignment of 73 sequences. Residues are colored by identity and conserved residues listed in Supplementary Table 4 are boxed in red. Structural regions as identified from our reconstruction are depicted above the alignment. Sequence alignment was performed using MAFFT and visualized with MView.[6,7] AcMNPV: *A. californica* MNPV; BmNPV: *Bombyx mori* NPV; HaNPV: *H. armigera* NPV; SeNPV: *S. exigua* NPV; PxGV: *P. xylostella* GV; SlGV: *S. litura* GV; NsNPV: *N. sertifer* NPV; CnNPV: *C. nigripalpus* NPV.

Supplementary Fig. 11: Phylogenetic tree of baculoviral VP39 calculated from 73 sequences.

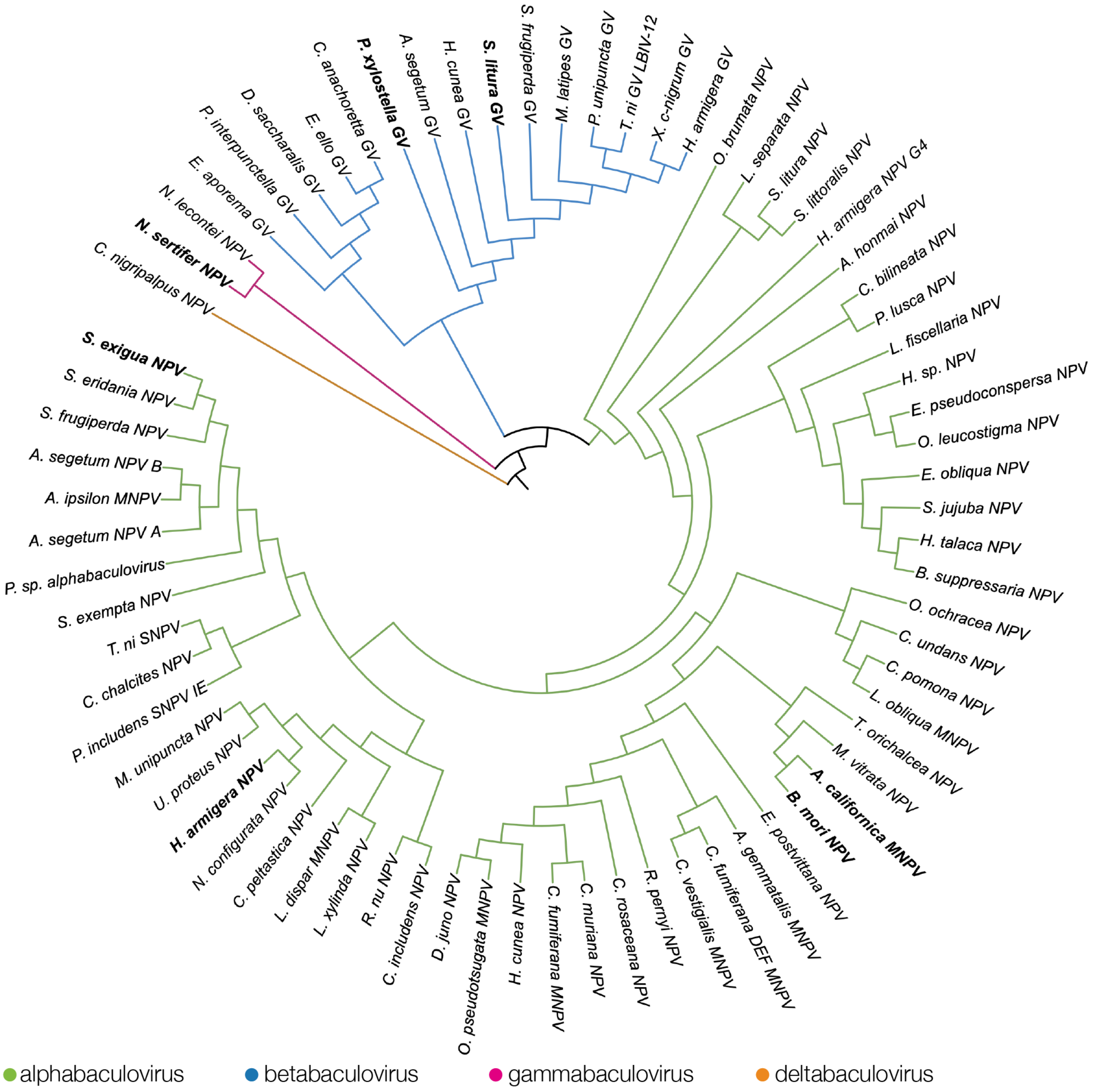

Viral species sequences that were used for helical reconstruction (AcMNPV) and for structure predictions are highlighted in bold. Branches are colored according to viral genera: alphabaculovirus (green), betabaculovirus (blue), gammabaculovirus (pink), deltabaculovirus (orange). The phylogenetic tree was calculated with Jalview[8] using the Neighbor Joining algorithm with a BLOSUM62 substitution matrix[9] and visualized using

the Interactive Tree of Life iTOL v. 6.7.4[10]. Branch lengths are ignored in the tree representation.

Supplementary Fig. 12: Confidence depiction for baculoviral VP39 dimer model predictions by AlphaFold2.

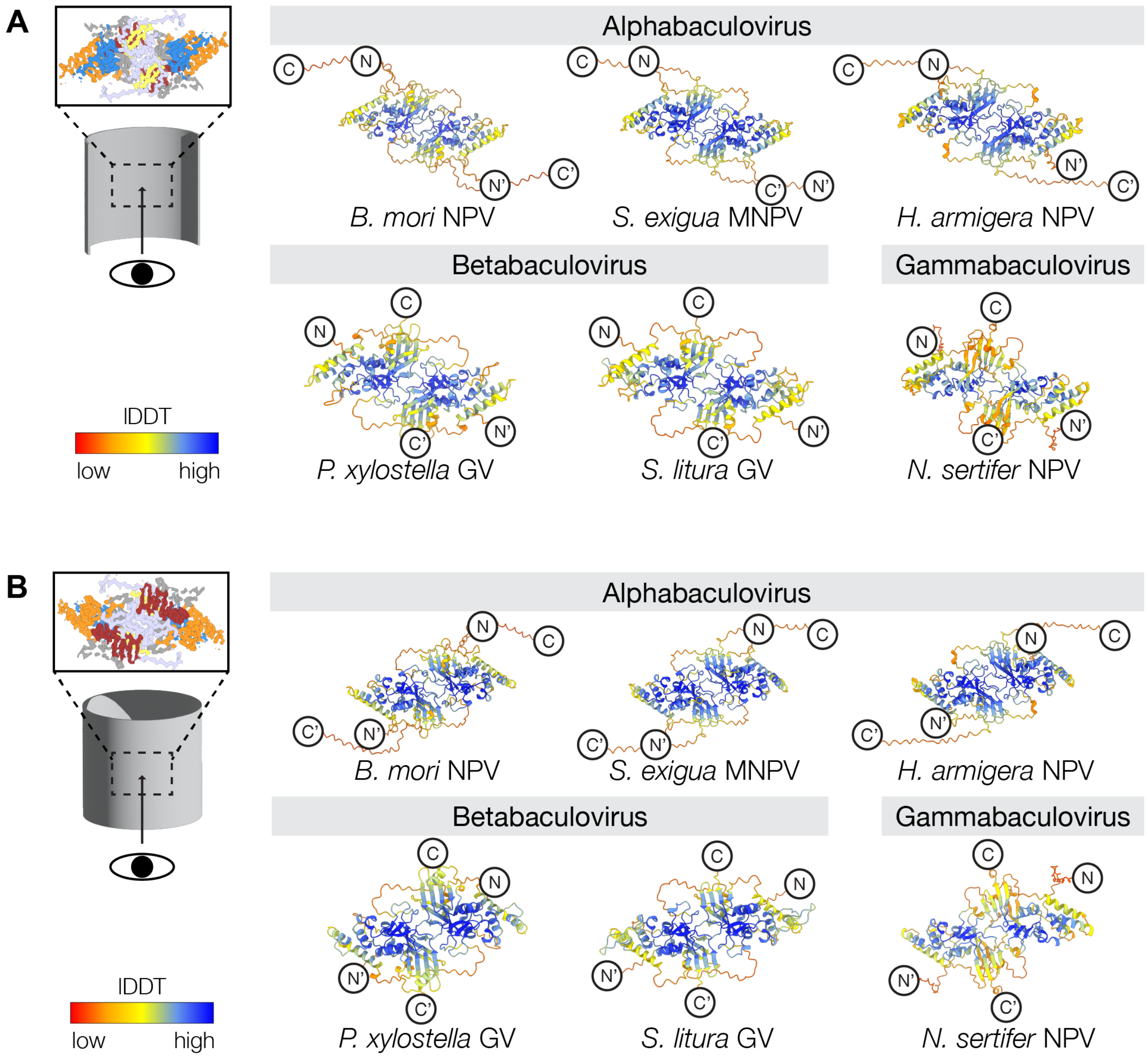

(A) Luminal side of baculoviral predicted VP39 dimer models. The structures are colored by prediction confidence from low (red) to high (blue), as indicated by the lDDT color bar. Prediction confidence is expressed through an lDDT-score, which is assigned a color[11]. Notably, prediction for the extended termini and inter-dimer stretches of the lasso loop are low.

(B) Exterior side of baculoviral VP39 dimer models, colored by prediction confidence.

NPV: nucleopolyhedrovirus; GV: granulovirus; lDDT: local Distance Difference Test.

Supplementary Fig. 13: Electrostatic surface potential of baculoviral VP39 dimer predictions.

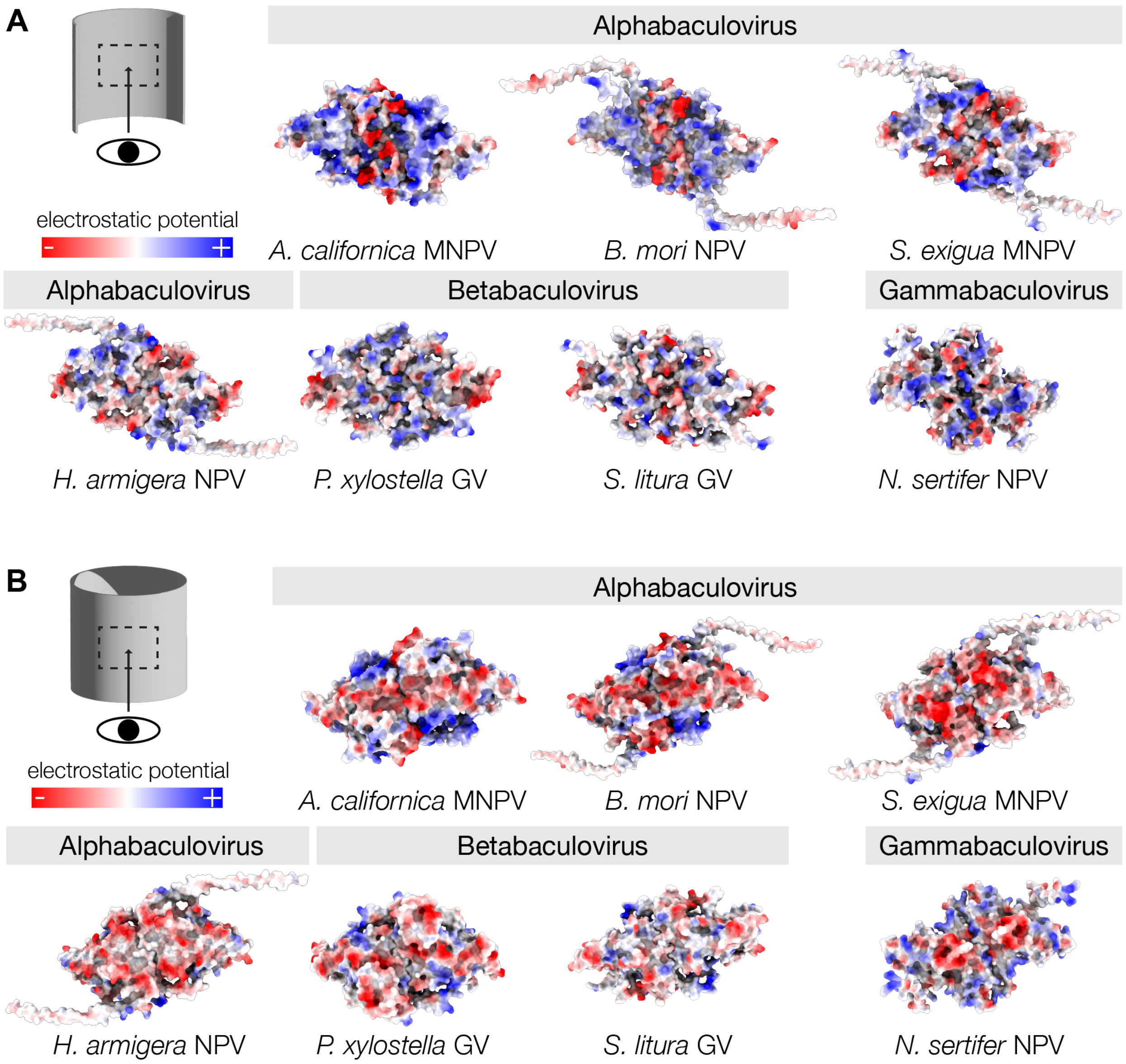

(A) Luminal view of VP39 dimer structure (AcMNPV) and AlphaFold2[12,13] predictions of the VP39 dimer. Electrostatic potential is colored from negative (red) to positive (blue). (B) Exterior view of VP39 dimer structure and predictions with electrostatic potential colored accordingly.

MNPV: multiple nucleopolyhedrovirus; NPV: nucleopolyhedrovirus; GV: granulovirus.

Supplementary Fig. 14: Comparison of observed and calculated power spectra of three non-flattened classes after supervised classification.

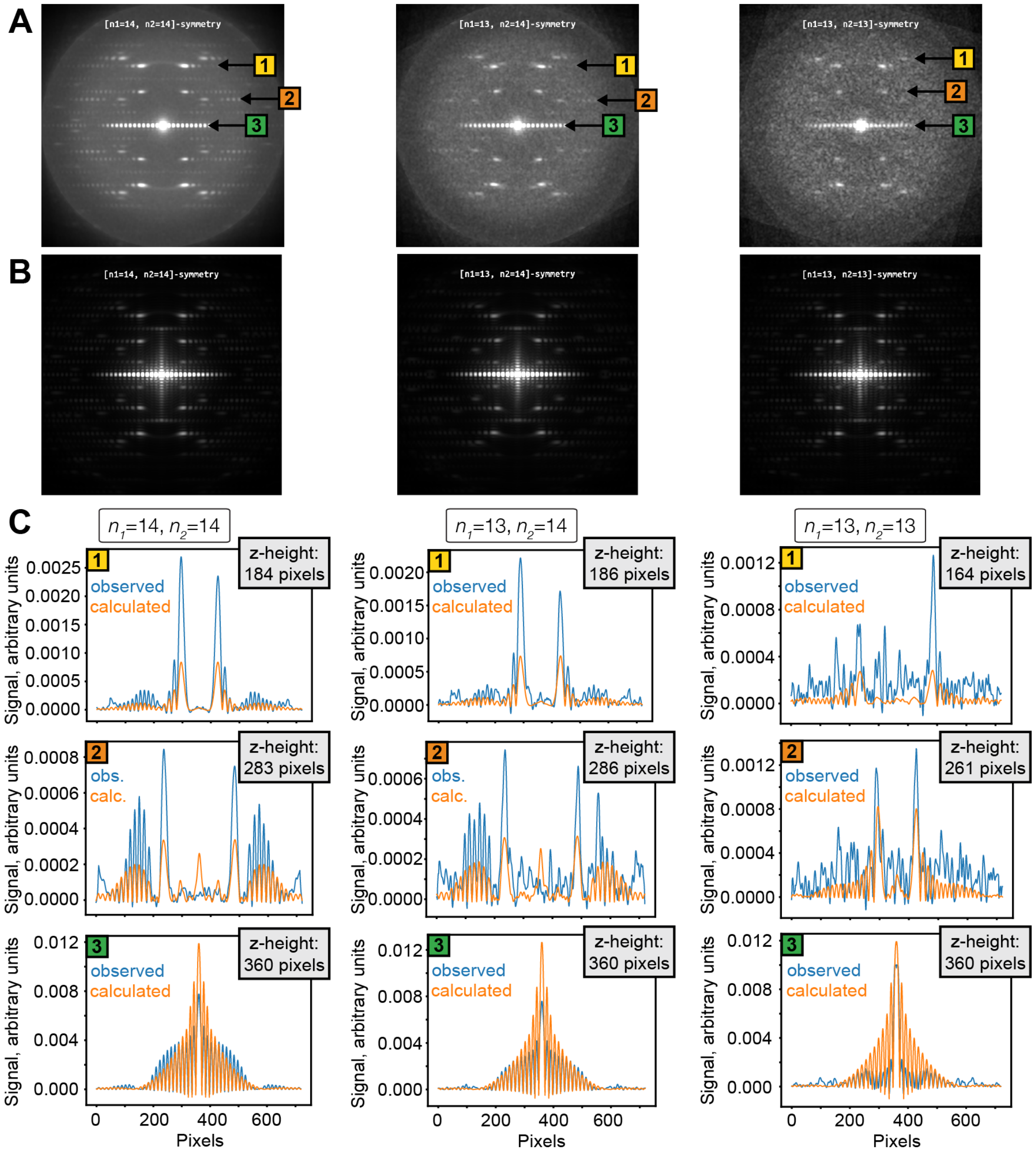

(A) Averaged power spectra of segments, which classified to a 3D reference with the indicated symmetry. Numbered arrows specify layer lines that were selected for direct comparison with the corresponding layer lines of calculated power spectra (shown in B)

in panel C. Segments were rotated according to the in-plane angle from the 3D alignment before calculating the power spectrum.

(B) Averaged power spectra calculated from 3D reference projections after orienting the 3D references according to the alignment parameters as observed for each segment. Projections were rotated according to the in-plane angle from the 3D alignment before calculating the power spectrum.

(C) Layer line profiles from power spectra of segments (shown in panel A; “observed”; blue lines) and calculated power spectra from reference projections (shown in panel B; “calculated”; orange lines) for prominent layer lines (denoted as 1, yellow; 2, red; 3, green in panel A) for each helical symmetry. For better comparison, layer line profiles were baseline corrected and arbitrarily scaled (linear scale factor). One pixel corresponds to a spatial frequency of 0.000166 $Å^{-1}$.

Supplementary Fig. 15: Classification analysis of the subparticle stack used for local reconstruction.

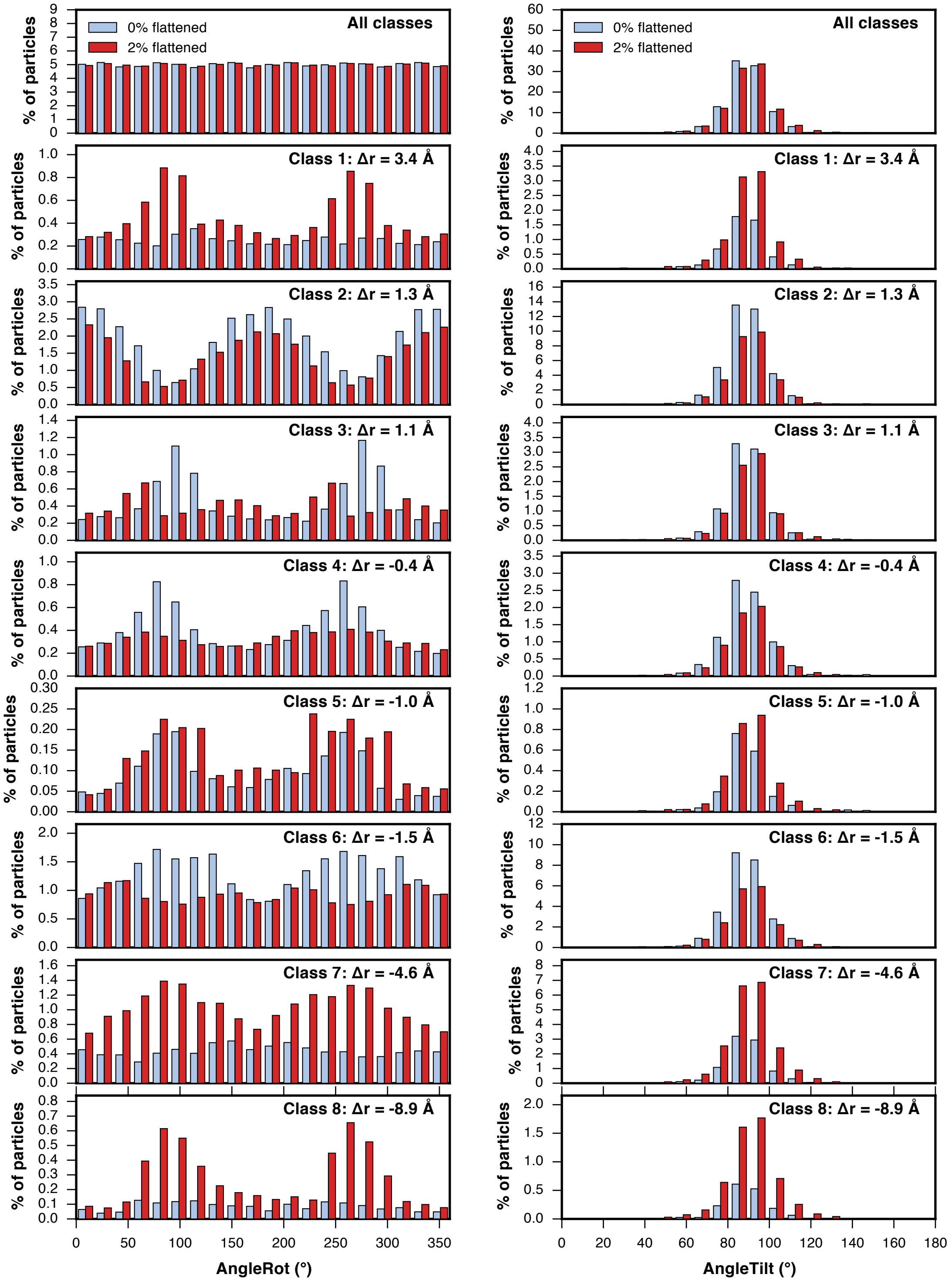

A subparticle stack extracted from 19,012 segments ([n_1=14, n_2=14]-symmetry and from non-flattened up to 2% flattened as determined by supervised classification) was classified into eight classes without changing the alignment. The plots show the distribution of the alignment angles (AngleRot, rotation around the helical axis; AngleTilt, out-of-plane rotation) for particles extracted from non-flattened (blue bars) and 2% flattened (red bars) segments, respectively. The top row shows the angles for particles from all classes, the following rows show each class individually with decreasing radial shifts from the helical axis (Δr). Classes with the largest radial shifts from the helical axis (classes 1, 7 and 8) show a substantial bias towards particles derived from 2% flattened segments. Source data are provided as a Source Data file.

Supplementary Fig. 16: Reconstructions of the [n_1=14, n_2=14]- and the [n_1=13, n_2=14]-classes after supervised classification.

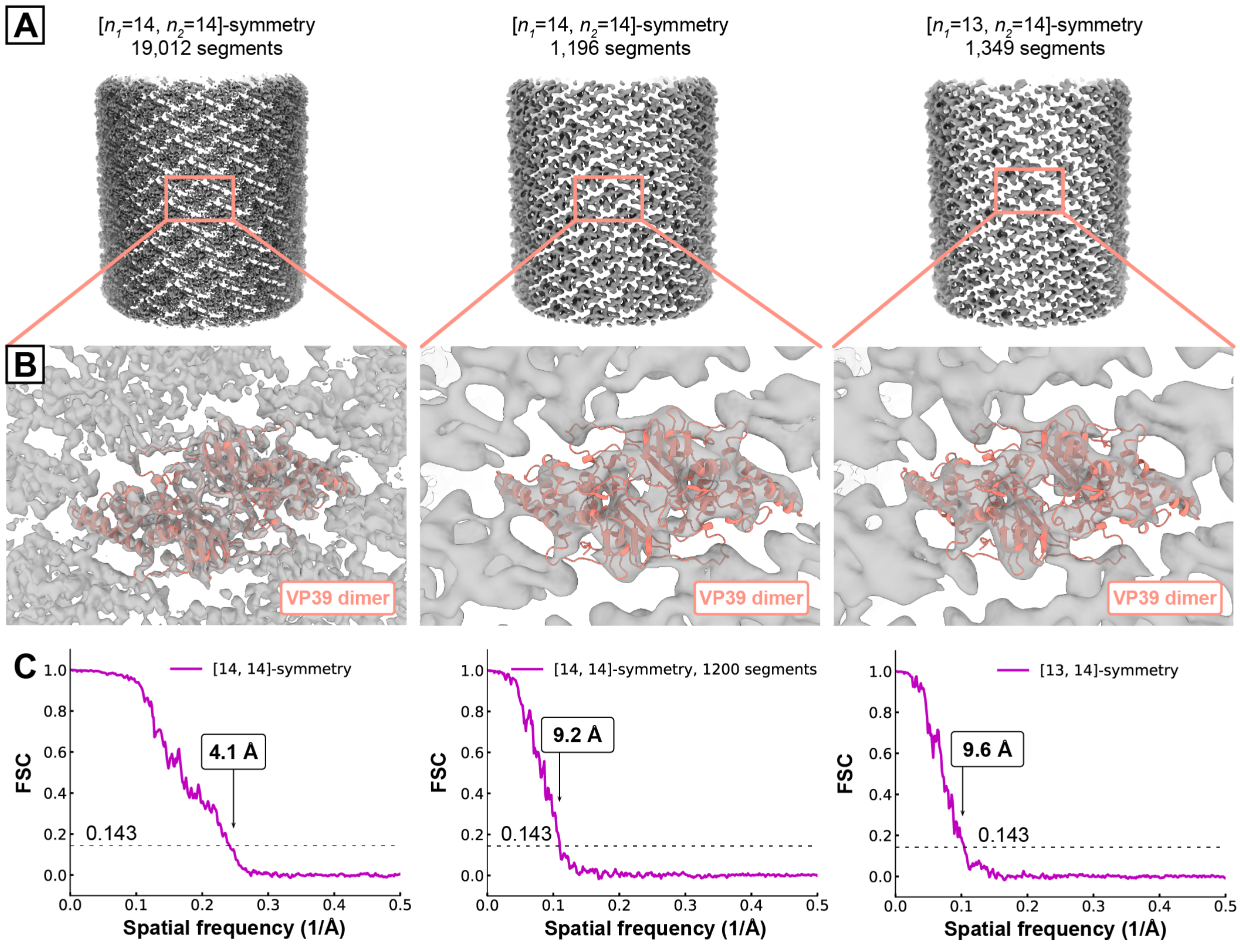

(A) Reconstructed volumes for segments, which classified to a 3D reference of 0-2% flattening with the indicated symmetry. Left: reconstruction of all 19,012 segments with [n_1=14, n_2=14]-symmetry at 4.1 Å using the simplified reconstruction protocol (see Methods). Center: reconstructed volume of 1,196 randomly selected segments with [n_1=14, n_2=14]-symmetry at 9.2 Å using the simplified reconstruction protocol to compare to reconstructions of helical symmetries with fewer segments. Right: reconstructed volumes of segments, which classified to a 3D reference of [n_1=13, n_2=14] at 9.6 Å (see Supplementary Table 5 for reconstruction statistics). Contrastingly to the main reconstruction protocol, only one cycle of the following steps was performed instead of

two cycles: segment alignment in cisTEM, reconstruction in C1, map symmetrization, and relion_ctf_refine. The main reconstruction protocol includes an additional helical symmetry search, map symmetrization in D14 and relion_motion_refine, which this simplified reconstruction protocol does not include. In the simplified reconstruction protocol applied here, map symmetrization is first performed using local symmetry (for protomers within a z_percentage of 0.3), followed by helical symmetry, whereas map symmetrization in the main reconstruction protocol is first performed using D14 rotational symmetry, followed by helical symmetry.

(B) Close-up of dimer region with the VP39 dimer fitted into the map.

(C) FSC curves calculated from half maps for each reconstruction. Source data are provided as a Source Data file.

Supplementary Fig. 17: The conserved glycine 276 is located at the type-ii axial inter-subunit interface.

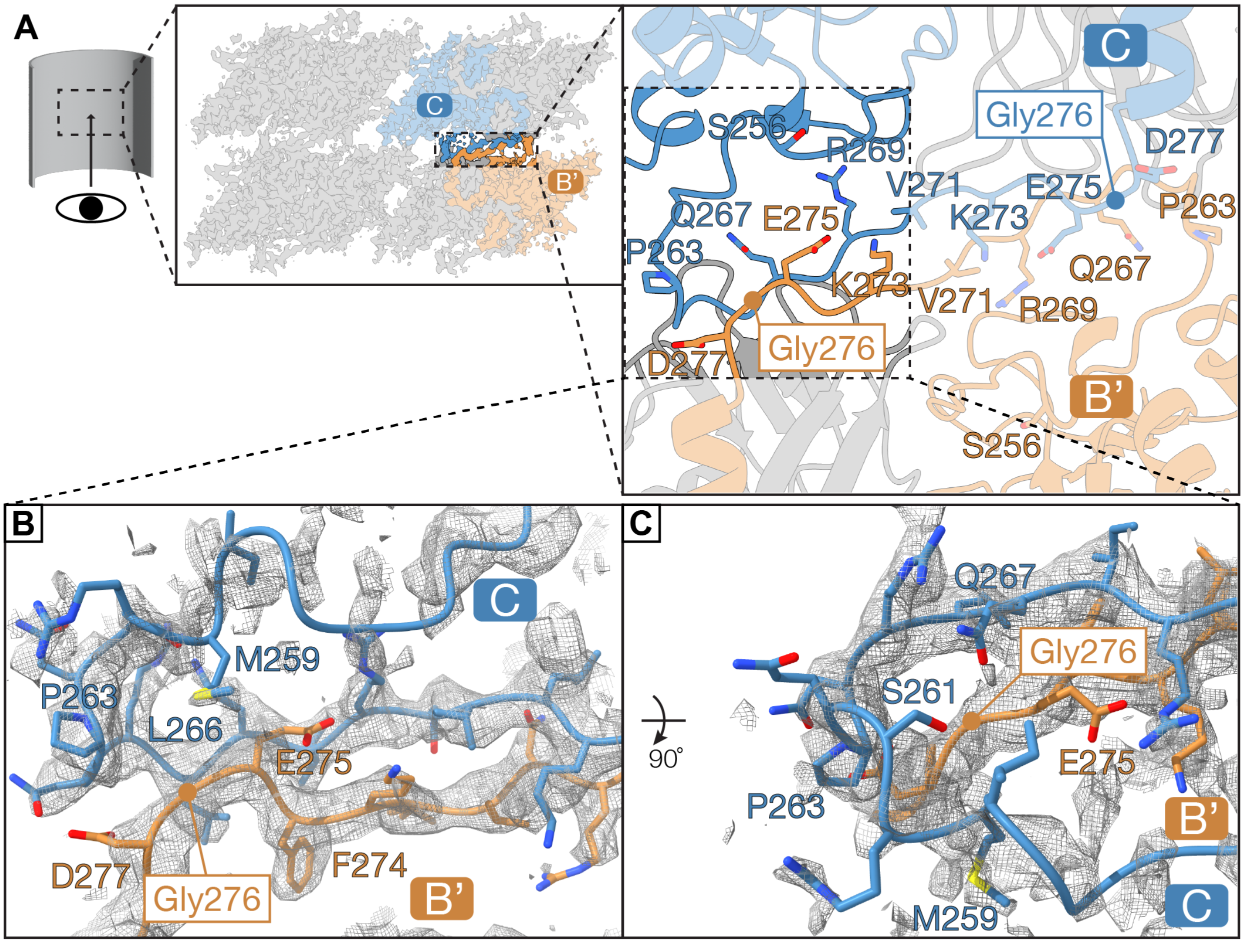

Morphologically aberrant nucleocapsids were observed in a mutagenesis screen with G276S[14].

(A) Schematic overview of the location of glycine 276, viewed from the luminal side of the capsid. Glycine 276 is nestled in between type-ii axial inter-subunit contacts on the lasso regions of two axially adjacent subunits B’ (ocher) and C (blue). All interface residues are labeled.

(B) Close-up view of the cryo-EM density in the vicinity of glycine 276 in monomer B’.

(C) Top view of the immediate environment surrounding glycine 276 in monomer B’.

Supplementary Fig. 18: Visualization of the candidate actin-binding residues 192-286.

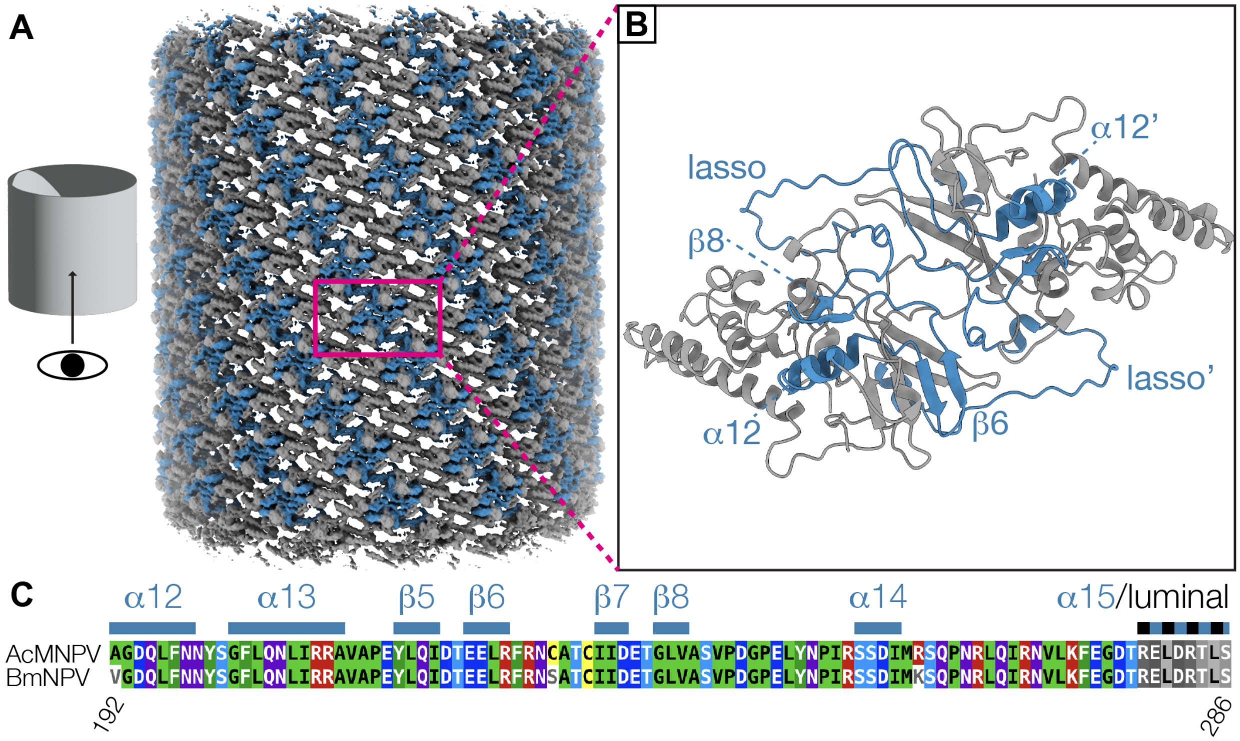

Residues 192-286 in BmNPV VP39 are required for nuclear actin polymerization[15].

(A) Cryo-EM map of the AcMNPV nucleocapsid with residues 192-286 colored in blue.

(B) Model of the AcMNPV dimer with residues 192-286 colored in blue.

(C) Sequence alignment of residues 192-286 of AcMNPV VP39 and BmNPV VP39 with residues colored by identity (96.8% sequence identity). The last 8 residues (279-286; gray-scale coloring) are facing the luminal side of the capsid. Secondary structure elements as identified from our reconstruction are depicted above the sequence alignment. Sequence alignment was performed using MAFFT[6] and visualized with MView[7].

Supplementary Table 1: Cryo-EM data collection and model statistics.

	AcMNPV VP39 nucleocapsid
**Data collection**	
Electron microscope	Titan Krios
Magnification	60606
Voltage (kV)	300
Defocus range (μm)[a]	0.4–1.9
Pixel size (Å)	0.825
Number of movies	45984
**Helical reconstruction, unbinned**	
Number of images	19,012
Box size (pixels)	912
Symmetry imposed	[n_1=14, n_2=14] [b], D14 followed by helical symmetry
Helical twist (˚)	-7.16
Helical rise (Å)	43.86
Map resolution (Å)[c]	3.6
**Local reconstruction**	
Number of images	209,286
Box size (pixels)	912
Symmetry imposed	C1
Map resolution (Å)[c]	3.2
**Model statistics**	
EMD accession identifier	EMD-41133
PDB accession identifier	8TAF
Refinement resolution (Å)	3.2
CC (mask)	0.78
Model composition	
Non-hydrogen atoms	39440
Protein residues	2464
Ligands	Zn: 8
B factors	
Protein (Å^2)	49.77 (mean)
Ligand (Å^2)	86.97 (mean)
R.m.s deviations	
Bond lengths (Å)	0.003
Bond angles (˚)	0.519
Validation	
MolProbity score	1.43
Clash score	3.12

Romater outliers (%)	0.4
Ramachandran plot	
Favored (%)	95.34
Allowed (%)	4.49
Outliers (%)	0.16

[a] Approximate range of underfocus.

[b] [n_1=14, n_2=14]-symmetry was applied by reconstruction in C1, then applying D14 symmetry followed by applying helical symmetry.

[c] Resolution where FSC between masked half maps drops below 0.143.

Supplementary Table 2: Analysis of the AcMNPV VP39 dimer interface.

Interface surface area and interaction types within the AcMNPV VP39 dimer were analyzed using the PDBePISA webserver[16].

Interface 1	Interface 2	Area [Å^2]	Hydrogen bonds	Salt bridges	ΔG_{sol}
Chain O	Chain J	3548.3	23	3	-35.5

List of hydrogen bonds comprising the AcMNPV VP39 dimer interface

Chain O	Chain J	Distance [Å]
Asp 44 [OD2]	Tyr 288 [HZ1]	1.53
Val 63 [H]	Glu 289 [O]	2.06
Val 63 [O]	Tyr 291 [H]	1.84
Phe 67 [O]	Tyr 250 [HH]	1.70
Asp 68 [OD2]	Phe 274 [H]	2.22
Asn 72 [HD21]	Phe 274 [O]	2.24
Arg 225 [HH11]	Pro 247 [O]	2.08
Arg 225 [O]	Tyr 250 [H]	1.87
Arg 227 [HH12]	Tyr 291 [OH]	1.94
Asp 245 [O]	Arg 225 [HH12]	2.04
Pro 247 [O]	Arg 225 [HH11]	1.98
Tyr 250 [H]	Arg 225 [O]	2.12
Tyr 250 [HH]	Phe 67 [O]	1.75
Arg 254 [HE]	Asp 70 [O]	1.79
Arg 254 [HH21]	Asp 70 [O]	1.97
Arg 254 [HH22]	Gln 73 [OE1]	2.23
Leu 272 [H]	Glu 69 [OE2]	2.40
Lys 273 [HZ2]	Asp 70 [OD2]	2.40
Phe 274 [H]	Asp 68 [OD2]	1.72
Phe 274 [O]	Asn 72 [HD21]	1.68
Tyr 288 [HH]	Asp 44 [OD2]	1.65
Glu 289 [O]	Val 63 [H]	2.32
Tyr 291 [H]	Val 63 [O]	1.79

List of salt bridges comprising the AcMNPV VP39 dimer interface

Chain O	Chain J	Distance [Å]
Lys 75 [NZ]	Asp 282 [OD1]	3.74
Arg 225 [NZ]	Asp 245 [OD2]	3.50
Lys 273 [NZ]	Asp 70 [OD2]	3.05

Supplementary Table 3: Analysis of the VP39 helical repeat unit contacts.

Interface surface area and interaction types between VP39 helical repeat units were analysed using the PDBePISA webserver[16].

LATERAL INTERACTIONS

Interface 1	Interface 2	Area [Å^2]	Hydrogen bonds	Salt bridges	ΔG_{sol}
Chain M	Chain J	605.8	1	0	-12.9
Chain P	Chain R	614.3	1	0	-14.6
	Average:	*610.0*			*-13.8*

List of hydrogen bonds at the lateral interface

Chain M	Chain J	Distance [Å]
Gln 146	Cys 169	2.10

Chain P	Chain R	Distance [Å]
Gln 146	Cys 169	1.87

TYPE-II AXIAL INTERACTIONS

Interface 1	Interface 2	Area [Å^2]	Hydrogen bonds	Salt bridges	ΔG_{sol}
Chain P	Chain O	703.0	10	2	-4.0
Chain M	Chain L	714.9	11	2	-8.5
	Average:	*709.0*			*-6.2*

List of hydrogen bonds at the type-II axial interface

Chain P	Chain O	Distance [Å]
Pro 263 [O]	Asp 277 [H]	2.25
Gln 267 [O]	Glu 275 [H]	1.95
Gln 267 [H]	Glu 275 [O]	2.36
Arg 269 [H]	Lys 273 [O]	2.50
Arg 269 [HE]	Glu 275 [OE2]	2.45
Lys 273 [H]	Arg 269 [O]	2.43
Lys 273 [O]	Arg 269 [H]	2.49
Glu 275 [H]	Gln 267 [O]	2.07
Glu 275 [O]	Gln 267 [H]	2.48
Asp 277 [H]	Pro 263 [O]	2.00

Chain M	Chain L	Distance [Å]
Pro 263 [O]	Asp 277 [H]	2.02
Gln 267 [O]	Glu 275 [H]	2.44
Gln 267 [H]	Glu 275 [O]	2.45
Arg 269 [O]	Lys 273 [H]	2.39
Val 271 [O]	Val 271 [H]	2.25
Val 271 [H]	Val 271 [O]	2.18
Lys 273 [H]	Arg 269 [O]	2.46
Lys 273 [O]	Arg 269 [H]	2.10
Glu 275 [H]	Gln 267 [O]	2.30
Glu 275 [OE2]	Arg 269 [HE]	2.16
Asp 277 [H]	Pro 263 [O]	2.37

List of salt bonds at the type-II axial interface

Chain P	Chain O	Distance [Å]
Arg 269 [NE]	Glu 275 [OE2]	3.12
Glu 275 [OE1]	Arg 269 [NE]	3.12

Chain M	Chain L	Distance [Å]
Arg 269 [NE]	Glu 275 [OE2]	3.86
Glu 275 [OE2]	Arg 269 [NE]	2.76

TYPE-I AXIAL INTERACTIONS

Interface 1	Interface 2	Area [Å^2]	Hydrogen bonds	Salt bridges	ΔG$_{sol}$
Chain Q	Chain O	439.1	4	0	-4.2
Chain P	Chain J	449.3	5	0	-3.3
Chain R	Chain M	450.4	4	0	-2.9
Chain N	Chain L	440.7	5	0	-3.1
	Average:	*444.9*			*-3.4*

List of hydrogen bonds at the type-I axial interface

Chain Q	Chain O	Distance [Å]
Ser 306 [O]	Arg 265 [HE]	2.28
Asn 308 [OD1]	Leu 266 [H]	2.21
Asn 308 [HD22]	Leu 266 [O]	2.27
Leu 311 [H]	Asn 264 [O]	2.35

Chain P	Chain J	Distance [Å]
Ser 306 [O]	Arg 265 [HE]	1.64
Ser 306 [O]	Arg 265 [HH21]	2.14
Glu 307 [OE1]	Asn 106 [HD22]	1.83
Asn 308 [OD1]	Leu 266 [H]	2.36
Leu 311 [H]	Asn 264 [O]	2.32

Chain R	Chain M	Distance [Å]
Ser 306 [O]	Arg 265 [HE]	1.91
Ser 306 [O]	Arg 265 [HH21]	2.16
Glu 307 [OE2]	Asn 106 [HD22]	2.04
Leu 311 [H]	Asn 264 [O]	2.43

Chain N	Chain L	Distance [Å]
Ser 306 [O]	Arg 265 [HE]	1.94
Ser 306 [O]	Arg 265 [HH21]	2.19
Glu 307 [OE2]	Asn 106 [HD22]	1.88
Asn 308 [OD1]	Leu 266 [H]	2.48
Leu 311 [H]	Asn 264 [O]	2.45

AXIAL-LATERAL INTERACTIONS

Interface 1	Interface 2	Area [Å^2]	Hydrogen bonds	Salt bridges	ΔG$_{sol}$
Chain P	Chain M	538.4	1	0	-5.0

List of hydrogen bonds at the axial-lateral interface

Chain P	Chain M	Distance [Å]
Glu 139 [OE2]	Phe 26 [H]	2.49

Supplementary Table 4: List of sequence-conserved residues of VP39.

73 VP39 sequences across baculoviruses (55 alphabaculoviruses, 15 betabaculoviruses, 2 gammabaculoviruses and 1 deltabaculovirus) were aligned using MAFFT[6] (see methods and Supplementary Fig. 10).

Residue[a]	Role
Cys 18	Zn^{2+} coordination
Phe 26[b]	Axial-lateral inter-subunit contact
Cys 36	Zn^{2+} coordination
Asp 44[c]	Intra-dimer contact
Cys 49	Zn^{2+} coordination
His 52	Zn^{2+} coordination
Cys 132	Candidate disulfide partner[d]
Glu 139[c,e]	Axial-lateral inter-subunit contact
Cys 169	Lateral inter-subunit contact, candidate disulfide partner[d]
Tyr 250[f]	Intra-dimer contact
Asn 264[b]	Type-I axial inter-subunit contact
Leu 266[b]	Type-I axial inter-subunit contact
Ile 268	unknown
Val 271	Type-II axial inter-subunit contact
Phe 274[c]	Intra-dimer contact
Glu 289[g]	Intra-dimer contact

[a]AcMNPV VP39 residue numbering

[b]conserved in alphabaculoviruses only

[c]conserved in alpha- and betabaculoviruses only

[d]Supplementary Fig. 9

[e]present as a glutamate or an aspartate

[f]Phe in gammabaculovirus

[g]Asp in gammabaculovirus

Supplementary Table 5: Statistics for reconstructions from segments after supervised classification for helical symmetries of three classes.

Symmetry	Number of segments	cisTEM score[b]	Resolution[c]	Resolution[d]
[14, 14]	19012	18.1	7.9	4.1
[14, 14][a]	1196	18.1	13.4	9.2
[13, 14]	1349	15.4	14.2	9.6
[13, 13]	224	10.8	62.7	N. A.

[a] 1,196 segments randomly selected from the 19,012 segments.

[b] Average alignment score after supervised classification.

[c] Resolution after reconstruction in C1 without symmetrization using alignment parameters from supervised classification.

[d] Resolution after alignment and relion_ctf_refine with symmetrization.

Supplementary Table 6: VP39 protein sequences used in this study.

VP39 Protein sequences used in this study are available in the UniProt database[17] and the NCBI reference sequence database RefSeq[18] under the following accession codes:

Baculovirus species	Accession code for VP39 protein sequence
Adoxophyes honmai NPV	Q80LM9
Agrotis ipsilon MNPV	B6D606
Agrotis segetum NPV A	YP_529756.1
Agrotis segetum NPV B	A0A0A7KR68
Antheraea pernyi NPV	Q1HH30
Anticarsia gemmatalis MNPV	A0A0S3J001
Autographa californica MNPV	P17499
Bombyx mori NPV	O92449
Buzura suppressaria NPV	W5VKH2
Catopsilia Pomona NPV	A0A172WZD1
Choristoneura fumiferana NPV	Q7TLR6
Choristoneura fumiferana DEF NPV	Q6VTQ7
Choristoneura murinana NPV	YP_008992160.1
Choristoneura rosaceana NPV	YP_008378418.1
Chrysodeixis chalcites NPV	Q4KSZ8
Chrysodeixis includens NPV	A0A5B8YRG4
Clanis bilineata NPV	Q0N423
Condylorrhiza vestigialis MNPV	A0A0B4UL98
Crytophlebia peltastica NPV	YP_010086942.1
Culex nigripalpus NPV (isolate Florida/1997)	Q919P5
Cychlophragma undans NPV	YP_010086669.1
Dione juno NPV	QDL56966.1
Ectropis obliqua NPV	A0EYX1
Epiphyas postvittana NPV	Q91GH6

Euproctis pseudoconspersa NPV	C3TWY4
Helicoverpa armigera MNPV	B7SUE9
Helicoverpa armigera NPV G4	NP_075147.1
Hemileuca sp. NPV	S5MQE4
Hyphantria cunea NPV	Q2NNW8
Hyposidra talaca NPV	YP_010086341.1
Lambdina fiscellaria NPV	A0A0E3Z6R9
Leucania separata NPV	Q0IL23
Lonomia obliqua MNPV	YP_009666419.1
Lymantria dispar MNPV	A0A140HQW4
Lymantria xylina NPV	YP_003517828.1
Mamestra configurata NPV B	Q8JM54
Maruca vitrata NPV	A1YRC9
Mythimna unipuncta NPV	YP_009666748.1
Neodiprion lecontei NPV (strain Canada)	Q6JP79
Neodiprion sertifer NPV	Q6JK71
Operophtera brumata NPV	YP_009552634.1
Orgyia leucostigma NPV	B0FDU5
Orgyia pseudotsugata MNPV	P17500
Oxyplax ochracea NPV	YP_009666582.1
Peridroma alphabaculovirus	A0A068LKA4
Perigonia lusca SNPV	A0A0M3WPB5
Pseudoplusia includens SNPV IE	A0A0B5A1E3
Rachiplusia nu NPV	QEI03677.1
Spodoptera eridania NPV	YP_010087081.1
Spodoptera exempta NPV	YP_010086493.1
Spodoptera exigua MNPV	Q9J862
Spodoptera frugiperda NPV	A1YJ67
Spodoptera littoralis NPV	YP_009505885.1
Spodoptera litura MNPV	Q9DWZ8

Sucra jujuba NPV	A0A097P905
Thysanoplusia orichalcea NPV	L0CLC9
Trichoplusia ni SNPV	Q461Y9
Urbanus proteus NPV	A0A161C6Y7
Agrotis segetum GV	YP_009513130.1
Clostera anachoreta GV	F4ZKV8
Diatraea saccharalis GV	A0A0R7EYZ4
Epinotia aporema GV	K4EQF1
Erinnyis ello GV	A0A097DAR9
Helicoverpa armigera GV	A9YMV5
Hyphantria cunea GV	QBQ01638.1
Mocis latipes GV	A0A162GVW7
Plodia interpunctella GV	A0A1L5JH15
Plutella xylostella GV	Q9DVV4
Pseudalatia unipuncta GV	B6S6Y4
Spodoptera frugiperda GV	A0A0C5AUX0
Spodoptera litura GV	A5IZT9
Trichoplusia ni GV LBIV-12	YP_009506175.1
Xestia c-nigrum GV	Q9PYT2

References

1. Grant, T., Rohou, A. & Grigorieff, N. cisTEM, user-friendly software for single-particle image processing. *eLife* **7**, e35383 (2018).
2. Tang, G. et al. EMAN2: An extensible image processing suite for electron microscopy. *Journal of Structural Biology* **157**, 38-46 (2007).
3. Winn, M.D. et al. Overview of the CCP4 suite and current developments. *Acta Crystallogr D Biol Crystallogr* **67**, 235-42 (2011).
4. Afonine, P.V. et al. New tools for the analysis and validation of cryo-EM maps and atomic models. *Acta Crystallographica Section D* **74**, 814-840 (2018).
5. Pettersen, E.F. et al. UCSF ChimeraX: Structure visualization for researchers, educators, and developers. *Protein Sci* **30**, 70-82 (2021).
6. Katoh, K., Rozewicki, J. & Yamada, K.D. MAFFT online service: multiple sequence alignment, interactive sequence choice and visualization. *Briefings in Bioinformatics* **20**, 1160-1166 (2017).
7. Madeira, F. et al. Search and sequence analysis tools services from EMBL-EBI in 2022. *Nucleic acids research* **50**, W276-W279 (2022).
8. Waterhouse, A.M., Procter, J.B., Martin, D.M.A., Clamp, M. & Barton, G.J. Jalview Version 2—a multiple sequence alignment editor and analysis workbench. *Bioinformatics* **25**, 1189-1191 (2009).
9. Saitou, N. & Nei, M. The neighbor-joining method: a new method for reconstructing phylogenetic trees. *Mol Biol Evol* **4**, 406-25 (1987).
10. Letunic, I. & Bork, P. Interactive Tree Of Life (iTOL) v5: an online tool for phylogenetic tree display and annotation. *Nucleic Acids Research* **49**, W293-W296 (2021).
11. Mariani, V., Biasini, M., Barbato, A. & Schwede, T. lDDT: a local superposition-free score for comparing protein structures and models using distance difference tests. *Bioinformatics* **29**, 2722-8 (2013).
12. Evans, R. et al. Protein complex prediction with AlphaFold-Multimer. *bioRxiv*, 2021.10.04.463034 (2022).

13. Jumper, J. et al. Highly accurate protein structure prediction with AlphaFold. *Nature* **596**, 583-589 (2021).

14. Katsuma, S. & Kokusho, R. A conserved glycine residue is required for proper functioning of a baculovirus VP39 protein. *J Virol* **91** (2017).

15. Zhang, J., Li, Y., Zhao, S. & Wu, X. Identification of a functional region in *Bombyx mori* nucleopolyhedrovirus VP39 that is essential for nuclear actin polymerization. *Virology* **550**, 37-50 (2020).

16. Krissinel, E. & Henrick, K. Inference of macromolecular assemblies from crystalline state. *J Mol Biol* **372**, 774-97 (2007).

17. Consortium, T.U. UniProt: the Universal Protein Knowledgebase in 2023. *Nucleic Acids Research* **51**, D523-D531 (2022).

18. O'Leary, N.A. et al. Reference sequence (RefSeq) database at NCBI: current status, taxonomic expansion, and functional annotation. *Nucleic Acids Res* **44**, D733-45 (2016).
