# Supplementary material for: Helical reconstruction of VP39 reveals principles for baculovirus nucleocapsid assembly
Source: Nat Commun. 2024 Jan 4;15:250. doi: 10.1038/s41467-023-44596-y (PMC10767040; doi:10.1038/s41467-023-44596-y)
Supplement: Supplementary file 3 — Description of Additional Supplementary Files [file 41467_2023_44596_MOESM3_ESM.pdf]

Description of Additional Supplementary Files

File Name: Supplementary Movie 1
Description: Animation of the deformation of the [n1=14, n2=14]-symmetry nucleocapsid at 0-9% flattening. The animation was created from ten 3D references of [n1=14, n2=14]-symmetry, which were created with wrapping vectors that infer increased tube flattening. The loading bar indicates the degree of flattening.

File Name: Supplementary Movie 2
Description: Animation of power spectra of the [n1=14, n2=14]-symmetry nucleocapsid at 0-9% flattening. (Left) Observed averaged power spectra of segments corresponding to the [n1=14, n2=14]- symmetry tube at 0-9% flattening. (Right) Calculated power spectra of back projections generated from the ten 3D references of [n1=14, n2=14]-symmetry at 0-9% flattening. The loading bar indicates the degree of flattening.

File Name: Supplementary Movie 3
Description: Fit between the observed and calculated averaged power spectra for the [n1=14, n2=14]-, [n1=13, n2=14]- and [n1=13, n2=13]-helical symmetry.

File Name: Supplementary Movie 4
Description: Difference between the power spectra from different helical symmetries for the [n1=14, n2=14]-, [n1=13, n2=14]- and [n1=13, n2=13]-helical symmetry.
