# Supplementary material for: Helical reconstruction of VP39 reveals principles for baculovirus nucleocapsid assembly
Source: Nat Commun. 2024 Jan 4;15:250. doi: 10.1038/s41467-023-44596-y (PMC10767040; doi:10.1038/s41467-023-44596-y)
Supplement: Supplementary file 8 — Reporting Summary [file 41467_2023_44596_MOESM8_ESM.pdf]

Corresponding author(s): Luke H. Chao

Last updated by author(s): 12/8/2023

Reporting Summary

Nature Portfolio wishes to improve the reproducibility of the work that we publish. This form provides structure for consistency and transparency in reporting. For further information on Nature Portfolio policies, see our Editorial Policies and the Editorial Policy Checklist.

Statistics

For all statistical analyses, confirm that the following items are present in the figure legend, table legend, main text, or Methods section.

n/a	Confirmed	
☐	☒	The exact sample size (*n*) for each experimental group/condition, given as a discrete number and unit of measurement
☐	☒	A statement on whether measurements were taken from distinct samples or whether the same sample was measured repeatedly
☒	☐	The statistical test(s) used AND whether they are one- or two-sided<br>*Only common tests should be described solely by name; describe more complex techniques in the Methods section.*
☒	☐	A description of all covariates tested
☒	☐	A description of any assumptions or corrections, such as tests of normality and adjustment for multiple comparisons
☐	☒	A full description of the statistical parameters including central tendency (e.g. means) or other basic estimates (e.g. regression coefficient) AND variation (e.g. standard deviation) or associated estimates of uncertainty (e.g. confidence intervals)
☒	☐	For null hypothesis testing, the test statistic (e.g. *F*, *t*, *r*) with confidence intervals, effect sizes, degrees of freedom and *P* value noted<br>*Give P values as exact values whenever suitable.*
☒	☐	For Bayesian analysis, information on the choice of priors and Markov chain Monte Carlo settings
☒	☐	For hierarchical and complex designs, identification of the appropriate level for tests and full reporting of outcomes
☒	☐	Estimates of effect sizes (e.g. Cohen's *d*, Pearson's *r*), indicating how they were calculated

Our web collection on statistics for biologists contains articles on many of the points above.

Software and code

Policy information about availability of computer code

Data collection	Serial EM (v3.7.1-64bit): Collection of images and movies from electron microscopes.<br>Reference: Mastronarde, D. N. Automated electron microscope tomography using robust prediction of specimen movements. J. Struct. Biol. 152, 36-51, doi:10.1016/j.jsb.2005.07.007 (2005).
Data analysis	MotionCor2 (vl.2.6): Alignment of movie frames.<br>Reference: Zheng, S. Q. et al. MotionCor2: anisotropic correction of beam-induced motion for improved cryo-electron microscopy. Nat. Methods 14, 331-332, doi:10.1038/nmeth.4193 (2017)<br><br>CtfFind4.1 (v4.1): CTF parameter estimation from electron micrographs.<br>Reference: Rohou, A. & Grigorieff, N. CTFFIND4: Fast and accurate defocus estimation from electron micrographs. J Struct Biol 192, 216-21 (2015)<br><br>CCP4 suite: software suite for biomolecular structure determination<br>Reference: Winn, M.D. et al. Overview of the CCP4 suite and current developments. Acta Crystallogr D Biol Crystallogr 67, 235-42 (2011)<br><br>EMAN2 (v2.22, https://blake.bcm.edu/emanwiki/EMAN2): EMAN2 is a broadly based greyscale scientific image processing suite with a primary focus on processing data from transmission electron microscopes.<br>Reference: Bell, J.M., Chen, M., Baldwin, P.R. & Ludtke, S. J. High resolution single particle refinement in EMAN2.1. Methods 100, 25-34, doi :10.1016/j.ymeth.2016.02.018 (2016)

Relion (v4.0): Cryo-EM data processing software.
Reference: Scheres, S. H. RELION: implementation of a Bayesian approach to cryo-EM structure determination. J. Struct. Biol. 180, 519-530, doi :10.1016/j.jsb.2012.09.006 (2012)

CryoSparc: Punjani, A., Rubinsstein, J.L., Fleet, D.J. & Brubaker, M.A. cryoSPARC: algorithms for rapid unsupervised cryo-EM structure determination. Nature Mathods 14, 290-296 (2017)

PyHi: Zhang, X. Python-based Helix Indexer: A graphical user interface program for finding symmetry of helical assembly through Fourier–Bessel indexing of electron microscopic data. Protein Science 31, 107-117 (2022)

cisTEM (refine3d version 1.01, reconstruct3d version 1.02, https://cistem.org): cisTEM is user-friendly software to process cry-EM images of macromolecular complexes and obtain high-resolution 3D reconstructions from them.
Reference: Grant, T., Rohou, A. & Grigorieff, N. cisTEM, user-friendly software for single-particle image processing. eLife 7, e35383 (2018)

SciPy (v1.8.0): SciPy provides algorithms for optimization, integration, interpolation, eigenvalue problems, algebraic equations, differential equations, statistics and many other classes of problems.
Reference: Virtanen, P., Gommers, R., Oliphant, T.E., Haberland, M., Reddy, T., Cournapeau, D., Burovski, E., Peterson, P., Weckesser, W., Bright, J., et al. (2020). SciPy 1.0: fundamental algorithms for scientific computing in Python. Nat. Methods 17, 261-272

NumPy: Harris, C.R. et al. Array programming with NumPy. Nature 585, 357-362 (2020)

BioPython (v1.78, https://biopython.org): Biopython is a set of freely available tools for biological computation written in Python by an international team of developers.
Reference: Cock, P. J. et al. Biopython: freely available Python tools for computational molecular biology and bioinformatics. Bioinformatics 25, 1422-1423, doi:10.1093/bioinformatics/btp163 (2009)

IMOD (v4.9.10, https://bio3d.colorado.edu/imod/): IMOD is a set of image processing, modeling and display programs used for tomographic reconstruction and for 3D reconstruction of EM serial sections and optical sections.
Reference: Kremer, J.R., Mastronarde, D.N. & McIntosh, J.R. Computer visualization of three-dimensional image data using IMOD. J Struct Biol 116, 71-6 (1996)

ChimeraX: Pettersen EF, Goddard TD, Huang CC, Meng EC, Couch GS, Croll TI, Morris JH, Ferrin TE. UCSF ChimeraX: Structure visualization for researchers, educators, and developers. Protein Science, 30, 70-82 (2021)

PHENIX (v1.17.1-3660, https://www.phenix-online.org): PHENIX is a software suite for the automated determination of molecular structures using X-ray crystallography and other methods.
References: Afonine, P.V. et al. Real-space refinement in PHENIX for cryo-EM and crystallography. Acta Crystallographica Section D 74, 531-544 (2018). Afonine, P.V. phenix.mtriage: a tool for analysis and validation of cryo-EM 3D reconstructions. Computational Crystallography Newsletter 8, 25 (2017).

MolProbity (v4.5, http://molprobity.biochem.duke.edu): Molecular structure validation.
Reference: Williams, C.J. et al. MolProbity: More and better reference data for improved all-atom structure validation. Protein Sci 27, 293-315 (2018)

PyMOL (v2.5, The PyMOL Molecular Graphics System, Version 2.1 Schrodinger, LLC): Molecular graphics visualization

AlphaFold2: Protein structure prediction
Reference: Jumper, J. et al. Highly accurate protein structure prediction with AlphaFold. Nature 596, 583-589 (2021)

AlphaFold multimer: Protein structure prediction for multimeric sequences
Evans, R. et al. Protein complex prediction with AlphaFold-Multimer. bioRxiv, 2021.10.04.463034 (2022)

ColabFold: ColabFold offers accelerated prediction of protein structures and complexes by combining the fast homology search of MMseqs2 with AlphaFold2 or RoseTTAFold.
Reference: Mirdita, M. et al. ColabFold: making protein folding accessible to all. Nat Methods 19, 679-682 (2022)

COSMIC2: an easy, web-based, science gateway to simplify cryo-EM data analysis using a standardized workflow
Reference: Cianfrocco, M.A., Wong-Barnum, M., Youn, C., Wagner, R. & Leschziner, A. COSMIC2: A Science Gateway for Cryo-Electron Microscopy Structure Determination. in Proceedings of the Practice and Experience in Advanced Research Computing 2017 on Sustainability, Success and Impact Article 22 (Association for Computing Machinery, New Orleans, LA, USA, 2017)

local Distance Difference Test: a superposition-free score that evaluates local distance differences of all atoms in a model
Reference: Mariani, V., Biasini, M., Barbato, A. & Schwede, T. lDDT: a local superposition-free score for comparing protein structures and models using distance difference tests. Bioinformatics 29, 2722-8 (2013)

Jalview (v.2.11.2.6): a multiple sequence aligment editor
Reference: Waterhouse, A.M., Procter, J.B., Martin, D.M.A., Clamp, M. & Barton, G.J. Jalview Version 2—a multiple sequence alignment editor and analysis workbench. Bioinformatics 25, 1189-1191 (2009)

Neighbor-joining: method to reconstruct phylogenetic trees.

Reference: Saitou, N. & Nei, M. The neighbor-joining method: a new method for reconstructing phylogenetic trees. Mol Biol Evol 4, 406-25 (1987)

MAFFT (v7.471, https://mafft.cbrc.jp/alignment/software/): MAFFT is a multiple sequence alignment program for unix-like operating systems. Reference: Katoh, K., Misawa, K., Kuma, K. & Miyata, T. MAFFT: a novel method for rapid multiple sequence alignment based on fast Fourier transform. Nucleic Acids Res. 30, 3059-3066, doi:10.1093/nar/gkf436 (2002) and Katoh, K., Rozewicki, J. & Yamada, K.D. MAFFT online service: multiple sequence alignment, interactive sequence choice and visualization. Briefings in Bioinformatics 20, 1160-1166 (2017)

PISA (v2.1.2): Protein Interactions, Surfaces and Assemblies (PISA).
Reference: Krissinel, E., and Henrick, K. (2007). Inference of macromolecular assemblies from crystalline state. J Mol Biol 372, 774-797

ConSurf (v2016, https://consurf.tau.ac.il): Positional conservation scores from the multiple sequence alignments.
Reference: Ashkenazy, H., Abadi, S., Martz, E., Chay, O., Mayrose, I., Pupko, T., and Ben-Tal, N. (2016). ConSurf 2016: an improved methodology to estimate and visualize evolutionary conservation in macromolecules. Nucleic Acids Res 44, W344-350

ITOL: Letunic, I., Bork, P. Interactive Tree of life (iTOL) v5: an online tool for phylogenetic tree siplay and annotation. Nucleic Acids Res, 49, W293-W296 (2021)

CODE AVAILABILITY
The software programs used to generate and analyze the data of this study are publicly available. Custom-written Python scripts used to run the programs and analyze data are available from the corresponding author upon request.

For manuscripts utilizing custom algorithms or software that are central to the research but not yet described in published literature, software must be made available to editors and reviewers. We strongly encourage code deposition in a community repository (e.g. GitHub). See the Nature Portfolio guidelines for submitting code & software for further information.

Data

Policy information about availability of data

All manuscripts must include a data availability statement. This statement should provide the following information, where applicable:

- Accession codes, unique identifiers, or web links for publicly available datasets
- A description of any restrictions on data availability
- For clinical datasets or third party data, please ensure that the statement adheres to our policy

Data availability
The cryo-EM maps generated in this study have been deposited in the Electron Microscopy Data Bank under accession code EMD-41133 [https://www.ebi.ac.uk/emdb/EMD-41133]. The refined model coordinates generated in this study have been deposited in the Protein Data Bank under accession codes 8TAF [https://doi.org/10.2210/pdb8taf/pdb]. VP39 Protein sequences used in this study are available in the UniProt database and the NCBI reference sequence database RefSeq and their accession codes are provided in Supplementary Table 6. All data are available from the corresponding author upon request. Source data are provided as a Source Data file.

Research involving human participants, their data, or biological material

Policy information about studies with human participants or human data. See also policy information about sex, gender (identity/presentation), and sexual orientation and race, ethnicity and racism.

Reporting on sex and gender	N/A
Reporting on race, ethnicity, or other socially relevant groupings	N/A
Population characteristics	N/A
Recruitment	N/A
Ethics oversight	N/A

Note that full information on the approval of the study protocol must also be provided in the manuscript.

Field-specific reporting

Please select the one below that is the best fit for your research. If you are not sure, read the appropriate sections before making your selection.

[x] Life sciences [ ] Behavioural & social sciences [ ] Ecological, evolutionary & environmental sciences

For a reference copy of the document with all sections, see nature.com/documents/nr-reporting-summary-flat.pdf

Life sciences study design

All studies must disclose on these points even when the disclosure is negative.

Sample size	No explicit sample size calculations were performed to design the cryo-EM studies. The number of particles used for each final map is sufficient to obtain reliable classification and reconstruction results by cryo-EM (Scheres, S. H. RELION: implementation of a Bayesian approach to cryo-EM structure determination. J. Struct. Biol. 180, 519-530, doi:10.1016/ j.jsb.2012.09.006 (2012)).
Data exclusions	Bad particle images were manually excluded after 2D classification. This is the generally adopted practice in the cryo-EM field (Scheres, S. H.RELION: implementation of a Bayesian approach to cryo-EM structure determination. J. Struct. Biol. 180, 519-530, doi:10.1016/ j.jsb.2012.09.006 (2012). Grant, T., Rohou, A. & Grigorieff, N. cisTEM, user-friendly software for single-particle image processing. Elife 7, doi:10.7554, elife.35383 (2018)). The exclusion criteria were not pre-established, but self-evident from the appearance of the 2D class averages.
Replication	Three cryo-EM datasets were collected from three independently prepared samples, and evaluated independently, yielding consistent results. The final calculations presented in this paper are from a single cryo-EM dataset.
Randomization	For statistical validation, cryo-EM datasets were randomly split into two half for calculation of cross correlation coefficients (FSC curves) between two the half sets (see Methods section for details). 2D classification was started from a randomized class distribution.
Blinding	The analysis was performed on single cryo-EM datasets. Blinding of the cryo-EM analysis work-flow was not feasible. The investigators were not blinded to group allocation during data collection. There was no blinding in structural data analysis, because we are studying a specific virus assembly.

Reporting for specific materials, systems and methods

We require information from authors about some types of materials, experimental systems and methods used in many studies. Here, indicate whether each material, system or method listed is relevant to your study. If you are not sure if a list item applies to your research, read the appropriate section before selecting a response.

Materials & experimental systems

n/a	Involved in the study	
☒	☐	Antibodies
☐	☒	Eukaryotic cell lines
☒	☐	Palaeontology and archaeology
☒	☐	Animals and other organisms
☒	☐	Clinical data
☒	☐	Dual use research of concern
☒	☐	Plants

Methods

n/a	Involved in the study	
☒	☐	ChIP-seq
☒	☐	Flow cytometry
☒	☐	MRI-based neuroimaging

Eukaryotic cell lines

Policy information about cell lines and Sex and Gender in Research

Cell line source(s)	Spodoptera frugiperda Sf9 (catalog number 12659017, Thermo Fisher Scientific)
Authentication	The cell line was not authenticated.
Mycoplasma contamination	The cell line was not tested for Mycoplasma contamination.
Commonly misidentified lines (See ICLAC register)	SF9 cells are not listed in the ICLAC register of commonly misidentified cell lines.
